# NT2-derived astrocyte–neuron co-culture reflects physiological relevance and offers research validity

**DOI:** 10.1186/s11658-025-00765-z

**Published:** 2025-07-25

**Authors:** Sylwia Kedracka-Krok, Ewelina Fic, Zuzanna Cepil, Piotr Rybczyński, Agata Szlaga, Radosław Cacała, Sławomir Lasota, Anna Blasiak, Marta Dziedzicka-Wasylewska

**Affiliations:** 1 Faculty of Biochemistry, Biophysics and Biotechnology, Department of Physical Biochemistry, Jagiellonian University, Kraków, Poland; 2 Institute of Zoology and Biomedical Research, Jagiellonian University, Kraków, Poland; 3 Faculty of Biochemistry, Biophysics and Biotechnology, Department of Cell Biology, Jagiellonian University, Kraków, Poland

**Keywords:** Ntera-2, NT2, Human neurons, Human astrocytes, Co-culture, Proteome, Markers, Electrophysiology

## Abstract

**Background:**

Obtaining human neurons and astrocytes for in vitro studies presents a significant challenge owing to the complexity of replicating their development and functionality outside the human brain. The Ntera-2 cell line is a valuable source of human neurons and astrocytes in neuroscience research. However, differentiating Ntera-2 cells into neurons and astrocytes with all-*trans* retinoic acid is complicated by the lack of reliable markers to monitor differentiation stages effectively. This study aimed to characterize neuron-enriched and pure astrocyte cultures at two maturation stages and to compare these with the original Ntera-2 cells. Ntera-2 cells and NT2 cells are used interchangeably in this publication.

**Methods:**

Using an advanced proteomic approach, we assessed the protein composition and abundance of neuron and astrocyte co-cultures and discovered that the astrocytic protein profile in co-culture with neurons was more representative compared with that in pure astrocyte cultures. Additionally, electrophysiological studies were conducted to investigate the best astrocyte content for neuronal functionality.

**Results:**

Mass spectrometry-based analysis provided insights into over 9000 proteins, covering well-known protein markers, proteins unique to specific cell types, and differentially expressed proteins. Notably, differences in transcription factors, regulatory proteins, intermediate filaments, and proteins unique to early and mature astrocytes highlighted the distinct maturation, activation, and functional profiles of the various cells. These findings offer a straightforward tool for characterization and monitoring the differentiation process. Three weeks of maturation in pure culture yielded immature astrocytes; however, extending the maturation period to 6 weeks significantly altered the composition of the cellular proteome, indicating increased astrocyte maturity. Studies revealed a broader repertoire of astrocytic proteins in co-culture with neurons. Meanwhile, electrophysiological analyses demonstrated that a high content of astrocytes is essential for neuronal functional maturity.

**Conclusions:**

Astrocyte–neuron co-cultures offer a more accurate model of neural tissue than pure cultures, highlighting the complexity of cell maturation and providing insights for improving in vitro modeling of human neural development.

**Graphical Abstract:**

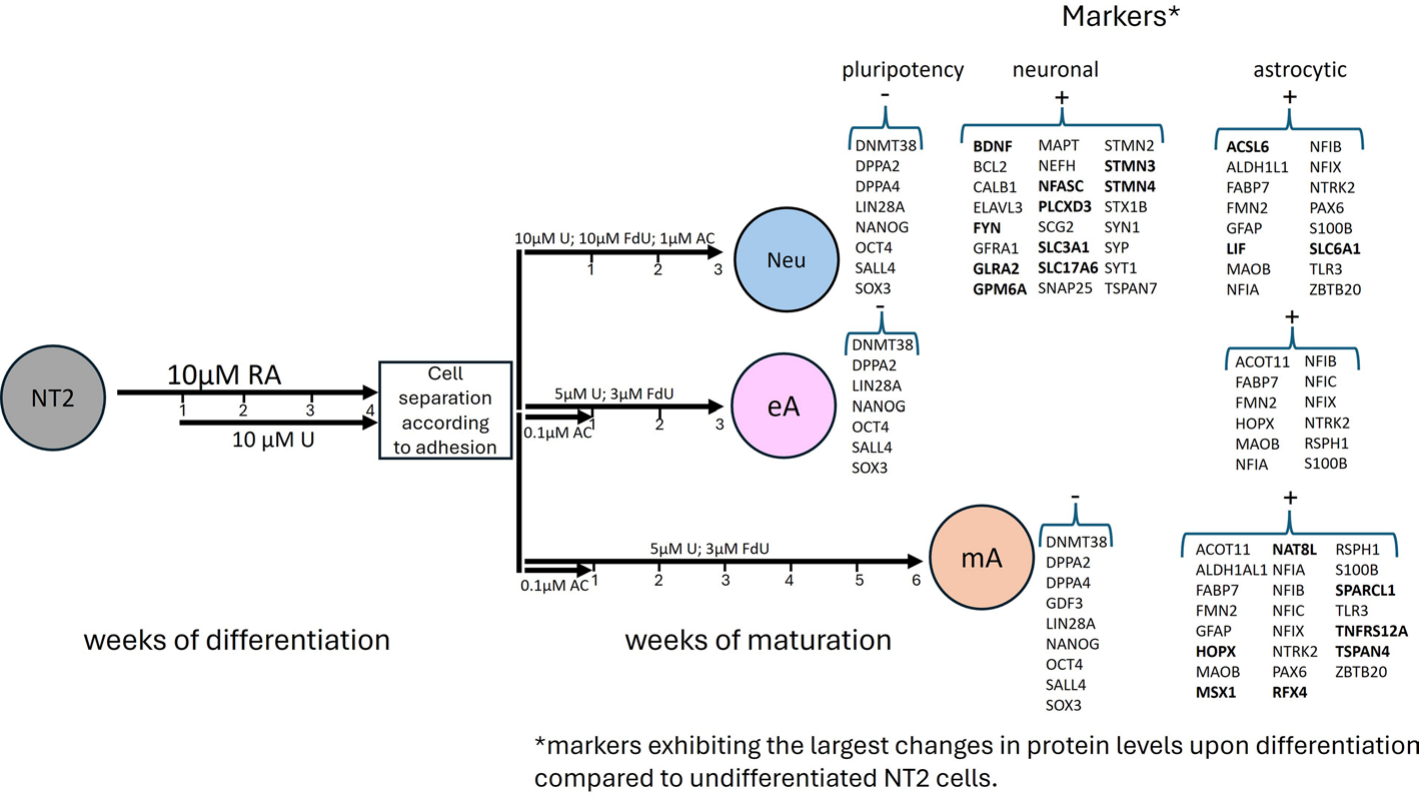

**Supplementary Information:**

The online version contains supplementary material available at 10.1186/s11658-025-00765-z.

## Introduction

NT2 cells (Ntera-2 cl.D1 cell line), derived by Andrews [1] from a human lung metastasis of a testicular teratocarcinoma, are pluripotent cells with an ability to generate cells from all three germ lineages in vitro [2, 3]. While Ntera-2-derived neurons serve as a valuable model in neurobiological studies, an equally interesting feature of the Ntera-2 cell line is that, following treatment with all-*trans* retinoic acid, a subpopulation of these cells differentiates into astrocytes. After separating early differentiated cell types, it becomes feasible to obtain pure cultures or cultures significantly enriched in neurons and/or astrocytes originating from the same genetic source, thus enabling researchers to carry out translational experiments on a more physiologically relevant co-culture of these cell types than using purely neuronal or astrocytic cell lines, such as SH-SY5Y or U-87MG, for example. This, in turn, facilitates more realistic modeling of interactions between neurons and astrocytes in the nervous system.

Human and rodent neuronal and glial cells display species-specific diversity in their cellular and molecular organization. In particular, human astrocytes differ from their mouse counterparts in terms of both morphology [4] and function. For instance, human astrocytes exhibit a greater susceptibility to oxidative stress compared with mouse astrocytes, primarily owing to differences in mitochondrial function and detoxification mechanisms. Additionally, mouse astrocytes, unlike their human counterparts, initiate a molecular response for neural repair under hypoxic conditions, while human astrocytes activate the antigen presentation pathway under inflammatory conditions [5].

Even though Ntera-2-derived neurons have been in use for over three decades, they are still not fully characterized. Ntera-2-derived astrocytes have received less attention in the literature, in terms of both their characteristics and their utility as a model of human astrocytes. Given the challenges associated with acquiring mature human astrocytes for experimental purposes owing to the limited availability of fresh healthy adult human brain tissue and considering that stem cell-derived human astrocytes frequently exhibit characteristics reminiscent of early developmental stages of these cells, Ntera-2-derived astrocytes represent a worthwhile alternative.

From several perspectives, a neuronal adherent culture has advantage over a suspension culture and organoids, including easier observation and manipulation of neurons in imaging, electrophysiology, and biochemical assays; higher homogeneity within the neuron population; improved experimental reproducibility; simpler culture scale-up; compatibility with certain assays such as those requiring neurite outgrowth observation; and greater suitability for long-term culturing. The use of adherent cultures of Ntera-2-derived neurons and astrocytes allows for effective separation and a subsequent in-depth study of these distinct cell types.

Typically, the evaluation of the cell differentiation processes relies on confirming the presence of only a few protein markers in the differentiated cells through immunocytostaining. Nevertheless, changes in cellular proteomes during differentiation and across different cell types can be significant.

In this article, we introduce the proteomic features of early (early A, eA) and mature (mature A, mA) astrocytes, and of a co-culture enriched with neurons (enriched N—60% of neurons and 40% of astrocytes, Neu) derived from the Ntera-2 cell line, and compare them with those of undifferentiated cells (NT2). We used label-free mass spectrometry-based approach with semi-absolute analysis to estimate protein copy numbers (the proteomic ruler approach) in the cell types studied as well as LFQ quantification to compare proteomes of differentiated and undifferentiated NT2 cells.

Through comprehensive protein profiling, we delineated the identity of neurons and their protein composition, confirming their intrinsic ability to generate action potentials. Furthermore, electrophysiological analysis validated their functional maturity and excitability.

Regarding astrocytes, we explored several critical protein groups, encompassing key regulatory proteins, transcription factors, intermediate filaments (specifically GFAP), and known protein markers. The results enabled us to confirm astrocyte identity and to better understand their maturation and activation status.

Our findings highlight the intricate interplay between astrocytes and neurons, deciphering key regulatory proteins and signaling pathways that mediate their reciprocal influence. Collectively, our study emphasizes the importance of co-culture models for accurately representing the complex dynamics of neural tissue.

## Materials and methods

### Cell culture and differentiation

Human pluripotent NT2 cells (ATCC; NTERA-2 cl.D1 cell line, ATCC-CRL-1973) were maintained as previously described [6–8], with certain modifications. NT2 cells were cultured in Dulbecco’s modified Eagle’s medium (DMEM, Sigma-Aldrich, USA)—high glucose (HG), including 10% fetal bovine serum (FBS, Thermo), 1.59 g/L sodium bicarbonate (Sigma-Aldrich, USA), and 1% penicillin/streptomycin (Sigma-Aldrich, USA). The cell culture was incubated at 37 °C with 5% CO_2_. The NT2 cells were further passaged with trypsin (trypsin–EDTA solution, Sigma-Aldrich, US) to reach the desired number.

For differentiation, 5 × 10^6^ cells were seeded in a 25-cm^2^ flask and treated with 10 μM all-*trans* retinoic acid (RA, Sigma-Aldrich, USA) three times a week over a period of 4 weeks. Starting from the second week of culturing with RA, 10 µM uridine was added three times a week for 3 weeks. The medium was changed twice a week. After RA treatment, the cells were collected using accutase (Accutase cell detachment solution, Sigma-Aldrich, USA) and plated with high density (13 × 10^6^ cells per 25-cm^2^ flask). The following day, cells were mechanically detached and the floating cells were replated again in a conditioned medium in a 25-cm^2^ flask at a density of 5 × 10^6^ cells (or on a microscope slide coated with poly-d-lysine at the corresponding density). Neuron maturation process was carried out in a conditioned medium supplemented three times a week with 1 µM cytosine arabinoside (AC, Sigma-Aldrich, USA), 10 µM uridine (U, Sigma-Aldrich, USA), and 10 µM fluorodeoxyuridine (FdU, Sigma-Aldrich, USA) for 3 weeks. The medium was changed twice a week.

Adherent cells were harvested using accutase and seeded into a 25-cm^2^ flask at a density of 5 × 10⁶ cells (or on a microscope slide coated with poly-d-lysine at the corresponding density). For the experiment involving early astrocytes (eA), the replating of adherent cells was carried out a day after the removal of neurons, with further cultivation proceeding for the additional 3 weeks. In the experiment with mature astrocytes (mA), the replating of adherent cells was performed 2 weeks post-neuron removal, and their cultivation continued for the next 4 weeks. The medium was changed twice weekly, but U and FdU were added three times a week at final concentrations of 5 µM and 3 µM, respectively. During the first week of astrocyte maturation (the week immediately following the completion of RA treatment), AC was also added three times a week to the maturing astrocytes at a final concentration of 0.1 µM.

Throughout the study, the neuron/astrocyte ratio in the culture flasks was determined by counting the cells in a Bürker chamber after mechanically and gently dislodging the less adherent neurons from the more adherent astrocytes, followed by gentle proteolytic separation of neurons from the spheres. The remaining adherent cells were harvested using a proteolytic enzyme and also counted in a Bürker chamber. On the basis of this experience, the neuron/astrocyte ratio in the wells of culture plates was estimated, as the cell numbers were too small for effective separation. Determining the ratio using bright-field or immunofluorescent images was challenging owing to the strong tendency of neurons to form spheres, making accurate cell counting difficult. Therefore, in the case of cultures conducted in culturing flasks, this method was not preferred. However, for coverslips—where mechanical separation of neurons and astrocytes is difficult—the neuron/astrocyte ratio was estimated by counting random fields under phase contrast microscopy assuming a spherical shape for the neuron aggregates.

The morphology of cells was monitored with an Olympus IX2-UCB phase-contrast inverted microscope.

### Immunocytochemistry

Undifferentiated or differentiated NT2 cells, either neurons or astrocytes, seeded on poly-d-lysine-coated coverslips, were fixed with 4% paraformaldehyde for approximately 15 min, then washed three times with PBS. These cells were permeabilized with 0.2% Triton X-100 in PBS for approximately 5 min, then washed three times with PBS, and nonspecific binding was blocked with 5% bovine serum albumin (BSA) in PBS for 1.5 h at room temperature (RT). Cells were incubated overnight at 4 °C with 20 μl of mixed mouse and rabbit primary antibody or single primary antibody per coverslip. Supplementary Table S1 lists antibodies suspended in 1% BSA and the dilution used. To visualize the cells, after three washes with PBS, they were stained with 20 μl of a mixture of Alexa Fluor 546- and 488-conjugated secondary antibodies (both diluted 1:500 in 3% BSA) per coverslip, for 2 h at RT in the dark. After two washes with PBS, cells were treated with 2 μg/ml Hoechst for 10 min to visualize DNA. Imaging was performed using a Leica DMI6000B microscope (Leica, Wetzlar, Germany), equipped with a DFC360FX CCD camera and a HCX PL APO 40/1.25 oil immersion objective. Exposure times and other imaging parameters were manually adjusted and preserved for particular marker proteins.

### Monitoring of cell morphology by videos

Videos were recorded using time-lapse imaging on a Leica DMI6000B inverted microscope equipped with a DFC360 FX CCD camera and an environmental chamber maintaining 37 °C and 5% CO_2_. Images were acquired using integrated modulation contrast (IMC) every 10 min over a period of 72 h. The videos were compressed using the H.264 codec and saved in .mov file format. The playback frame rate was 40 fps.

### Western blot

Cells were lysed with 100 mM Tris–HCl buffer at pH 7.5 and 2% SDS buffer, sonicated, and thermally denatured. After concentration measurement, samples containing 15 ug of protein were prepared. SDS-PAGE was performed in a Laemmli system (4/10% acrylamide/bis-acrylamide, 0.1% SDS gel, 25 mM Tris/200 mM glycine, 0.1% SDS running buffer, 45 min 100 V, 30 min 200 V). Proteins were transferred onto a PVDF membrane using CAPS transfer buffer (10 mM CAPS, pH 11, 10% methanol). Normalization was based on the total protein fluorescence method (20 min 0.1 M ruthenium(tris(bathophenanthrolinedisulfonate)) [RuBPS]); visualization was carried out using the ChemiDoc™ imaging system (BioRad, Auto Optimal). Membranes were blocked with a 5% albumin solution in TBS-T (20 mM Tris–HCl at pH 7.5, 150 mM NaCl, 0.1% Tween 20), then incubated for 1 h in primary anti-GFAP antibody solutions (Supplementary Table S1) followed by incubation with secondary antibody conjugates with Alexa Fluor Plus 647 (Supplementary Table S1). Fluorescent bands were visualized by ChemiDocTM Imaging System (BioRad, Auto Optimal). Statistical significance was tested with one-way ANOVA with Welch’s correction for unequal variances and the Games–Howell post hoc test.

### Proteomic analysis

#### Initial preparation of protein samples

For the extraction of total proteins, cell pellets were lysed using a lysis buffer consisting of 4% SDS (Fluka Chemie), 0.1 M DTT (Merck), and 0.1 M Tris (Roche) in Tris–HCl (BioShop) at pH 7.6. The lysates were then subjected to sonication for two cycles of 5 min each (320W, 30 s on/off) using a Bioruptor UCD-200 homogenizer (Diagenode). Subsequently, the samples were incubated at 95 °C for 5 min and centrifuged at 16,000 × *g* for 15 min at 20 °C. The resulting supernatants were collected and stored at –80 °C for further experimental steps.

#### Filter-aided sample preparation for LC–MS/MS analysis

Samples were prepared for global proteomic analysis by liquid chromatography-tandem mass spectrometry (LC–MS/MS) using the filter-assisted sample preparation (FASP) method described by Wisniewski et al. [9]. Initially, 100 µg of protein lysate was diluted in 300 µl of 8 M urea (Lab Empire) in 50 mM ammonium bicarbonate (ABC, Sigma) and then mixed with freshly added DTT (final concentration was 50 mM) by vortexing for 15 min. Subsequently, the samples were centrifuged (21,000 × *g*, 15 min, 20 °C) and applied on the 30-kDa cut-off filter (Vivacon 500, Sartorius Stedim Biotech GmbH). The next steps including alkylation, washing with the urea solution, as well as washing with 50 mM ABC were conducted according to protocol 1 derived from the FASP Protein Digestion Kit Use and Storage Instructions (Expendeon). Following washing, 1 µg of Lys-C protease (Promega) resuspended in ABC solution was added. After on-filter protein digestion (overnight, 37 °C), the resulting peptides were spun down (14,000 × *g*, 15 min, 20°C) into new tubes and subsequently washed out of the membrane two times with ABC solution (14,000 × *g*, 15 min, 20 °C). In the next step, 1 µg of trypsin (Promega) resuspended in ABC solution was added to the column. After overnight digestion at 37 °C, the peptides obtained were centrifuged (14,000 × *g*, 15 min, 20 °C) to new tubes and further washed out of the membrane according to the procedure used after Lys-C digestion. This process resulted in two distinct peptide mixtures for each sample, i.e., (1) peptides obtained after digestion with Lys-C protease and (2) peptides obtained after digestion with trypsin.

#### Fractionation of peptides

The peptides obtained were fractionated as described by Wisniewski et al. [10] via strong anion exchange (SAX) using tip columns packed with six layers of resin disks (Empore Anion-SR, 47 mm Extraction Disks, Supelco, Sigma-Aldrich). Peptides obtained after Lys-C digestion were separated into four fractions using Britton–Robinson universal buffers (BRUBs) at pH 11, 6, 4, and 2, while peptides obtained after trypsin digestion were separated into two fractions with BRUBs at pH 5 and 2. Then, the obtained fractions underwent purification using tip columns packed with three layers of C18 resin disks (Empore Octadecyl C18, 47 mm Extraction Disks, Supelco, Sigma-Aldrich) using the following protocol: precondition with methanol (JT Baker); washing with 60% acetonitrile (ACN, JT Baker)/1% acetic acid (JT Baker) solution; equilibration with 1% acetic acid; sample loading; washing with 1% acetic acid; elution with 60% ACN/1% acetic acid solution. Subsequently, the samples were vacuum-dried and further suspended in 2% ACN/0.05% trifluoroacetic acid (TFA, JT Baker) solution for further LC–MS/MS analysis.

#### LC–MS/MS acquisition

The peptides were analyzed using mass spectrometry (MS) employing a high-resolution Q-Exactive mass spectrometer (Thermo Scientific) coupled with an UltiMate 3000RS LC nanoSystem (Thermo Scientific). Initially, the peptides were loaded onto a C18 precolumn (Acclaim PepMap Nano trap Column, Thermo Scientific; ID 75 µm, length 20 mm, particle size 3 µm, pore size 100 Å) using a mobile phase of 2% ACN with 0.05% TFA at a flow rate of 5 µm/min. Subsequently, they were separated on a C18 analytical column (Acclaim PepMap RLSC C18, ThermoScientific; ID 75 µm, length 500 mm, particle size 2 µm, pore size 100 Å) employing a 210-min gradient of ACN ranging from 2% to 40% in 0.05% formic acid (JT Baker). Ionization of peptides occurred in a Digital PicoView 550 ion source (New Objective, Woburn, MA, USA). Mass spectrometry measurements utilized the top 12 method with full MS and MS/MS resolutions of 70,000 and 17,500, respectively.

#### LC–MS/MS data analysis

In the experiment, at least five biological replicates of each of the studied cell types were profiled. For undifferentiated NT2 culture, biological replicates encompassed cells from passage 12 to 21. The data obtained from LC–MS/MS were subsequently analyzed using MaxQuant version 2.1.4.0 [11]. Briefly, MaxQuant facilitated searches against the SwissProt database restricted to *Homo sapiens* taxonomy, which contained 20,404 sequences (as of 7 January 2023), and was supplemented with common protein contaminant sequences. The search parameters included: enzyme—trypsin; number of missed cleavages—2; static modification—carbamidomethylation (C); dynamic modifications—oxidation (M) and acetylation (protein N-terminus). Other software settings were left at default and included a false discovery rate (FDR) below 1% for peptide and protein identification.

Proteins that were present in at least 50% of the samples in a group, and simultaneously had a sum of sequenced peptides exceeding 130% of the number of samples in the group, were selected for GO enrichment analysis. This analysis, conducted on the identified proteins in the studied cell types using FunRICH [12], applied a *p*-value threshold with a Benjamini–Hochberg correction of less than 0.05.

Furthermore, a quantitative analysis was conducted using Perseus software version 2.0.11 [13]. Proteomic ruler quantification was performed as described in Ref. [14]. The comparison between cell types was based on log_2_-transformed LFQ intensities for proteins present in at least 70% of samples in total. Proteins that met a permutation-based FDR of 0.0001 in the ANOVA test, were significant in the post hoc Tukey test, and exhibited at least a twofold change in quantity between the compared groups were included in further analyses using Ingenuity Pathway Analysis (IPA, QIAGEN) software. Only canonical pathways that met the Benjamini–Hochberg correction with a significance level of 0.05 are presented in this paper.

#### Calcium response imaging of mA cells

Calcium response to stimulation and changes in Ca^2+^ concentration were monitored using confocal microscopy. mA astrocytes, which matured for 6 weeks on poly-d-lysine-coated coverslips were washed twice with a buffer containing (in mM): 140 NaCl, 4.8 KCl, 1 MgCl_2_, 10 HEPES, 1 CaCl_2_, and 10 glucose (pH 7.2).The cells were then incubated with the membrane-permeant fluorescent Ca^2+^-sensitive dye Fluo-4-AM for 30 min in the dark, followed by another wash with the same buffer. Just before measurement, the cells were stimulated with bath applied ATP (final concentration 10 µM), and the Ca^2+^ flow was observed under the microscope. Fluorescence intensity was monitored using a Zeiss LSM 900 confocal microscope, with excitation/emission filters set for excitation at 450–490 nm and emission at 500–550 nm, measured using an LD Plan-Neofluar 20x/0.4 Korr M27 objective. The exposure time was 400 ms, and the image size was 710.52 µm × 532.38 µm (1388 × 1040 pixels).

#### Electrophysiology of neuronal cells

During whole-cell patch-clamp electrophysiological recordings, NT2 cell lines were continuously perfused (1.1 ml/min) with carbogenated, warm (35 °C) artificial cerebrospinal fluid (ACSF), containing (in mM): 118 NaCl, 25 NaHCO_3_, 3 KCl, 1.2 NaH_2_PO_4_, 2 CaCl_2_, 1.3 MgSO_4_ and 10 glucose, (pH 7.4; osmolality 290–300 mOsmol/kg). Recording micropipettes were fabricated from borosilicate glass capillaries (7–9 MΩ; Sutter Instruments, Novato, CA, USA) using a horizontal puller (Sutter Instruments) and filled with a solution containing (in mM): 145 potassium gluconate, 2 MgCl_2_, 4 Na_2_ATP, 0.4 Na_3_GTP, 5 EGTA, 10 HEPES (pH 7.3; osmolality 290–300 mOsmol/kg), and biocytin (0.05%). The calculated liquid junction potential was +15 mV, and data were corrected for this value. All reagents for ACSF and intrapipette solution were purchased from Sigma-Aldrich (Darmstadt, Germany), apart from biocytin, which was purchased from Tocris Bioscience (Bristol, UK).

NT2 cells were visually identified under an Examiner D1 microscope (Zeiss, Oberkochen, Germany) equipped with video-enhanced infrared differential interference contrast. Cell-attached and whole-cell configurations were obtained using a negative pressure delivered by mouth suction. SEC 05LX amplifier (NPI, Tamm, Germany), Micro 1401 mk II (CED, Cambridge Electronic Design, Cambridge, UK) converter, and Signal and Spike2 software (CED) were used for signal recording and data acquisition. The recorded signal was low-pass filtered at 3 kHz and digitized at 20 kHz.

A cell was considered a functional neuron if at least one action potential (AP) was generated in response to the depolarizing current steps described later on, and only the properties of these cells were analyzed. Electrophysiological properties of NT2-derived neurons were analyzed using custom Spike2 and Signal (CED) as well as MATLAB (The MathWorks, Natick, MA, USA) scripts. Membrane properties and excitability were calculated from voltage responses to current or stimuli delivered from a membrane potential of −75 mV, sustained with continuous current injections. Excitability of the recorded neurons was quantified by calculating the number of spikes in response to incremental (10 to 140 pA, 10 pA increment, 500 ms) depolarizing current pulses. To assess the AP shape, the first action potential generated after depolarization with +80 pA 500-ms-long current step was taken. The AP threshold was assessed based on the voltage response to current ramp (0–1 nA, 1 s). In voltage clamp mode, voltage steps from −120 to + 10 mV (10 mV change, 500 ms pulse duration, delivered from a membrane potential of −75mV) were used to measure the *I*–*V* relationships of the steady-state current. Steady-state current density was calculated by dividing the current by the capacitance of a given neuron. The resting membrane potential was calculated from a 200-s recording at zero holding current. Passive membrane properties of the recorded neurons, including capacitance for calculating steady-state current density, were calculated on the basis of the voltage response to a hyperpolarizing current pulse (−40 pA, 500 ms).

Statistical analysis was performed using GraphPad Prism version 8.0.1 for Windows (GraphPad Software Inc., La Jolla, CA, USA). All data underwent outlier detection (ROUT method, *Q* = 1%), and outliers were eliminated from the analysis. Differences were considered statistically significant at *p* < 0.05. All tests were two-tailed. When data followed normal distribution (assessed by Anderson–Darling test), one-way ANOVA or two-way ANOVA were used; otherwise the nonparametric Kruskal–Wallis test was used, as specified in the “Results” section and in the figure descriptions. All values are provided as mean ± SD (for normally distributed data) or median with interquartile range (for data that did not pass normality tests).

## Results

### Differentiation of human NT2 cells into neurons and astrocytes—culture efficiency and purity

**Fig. 1:**
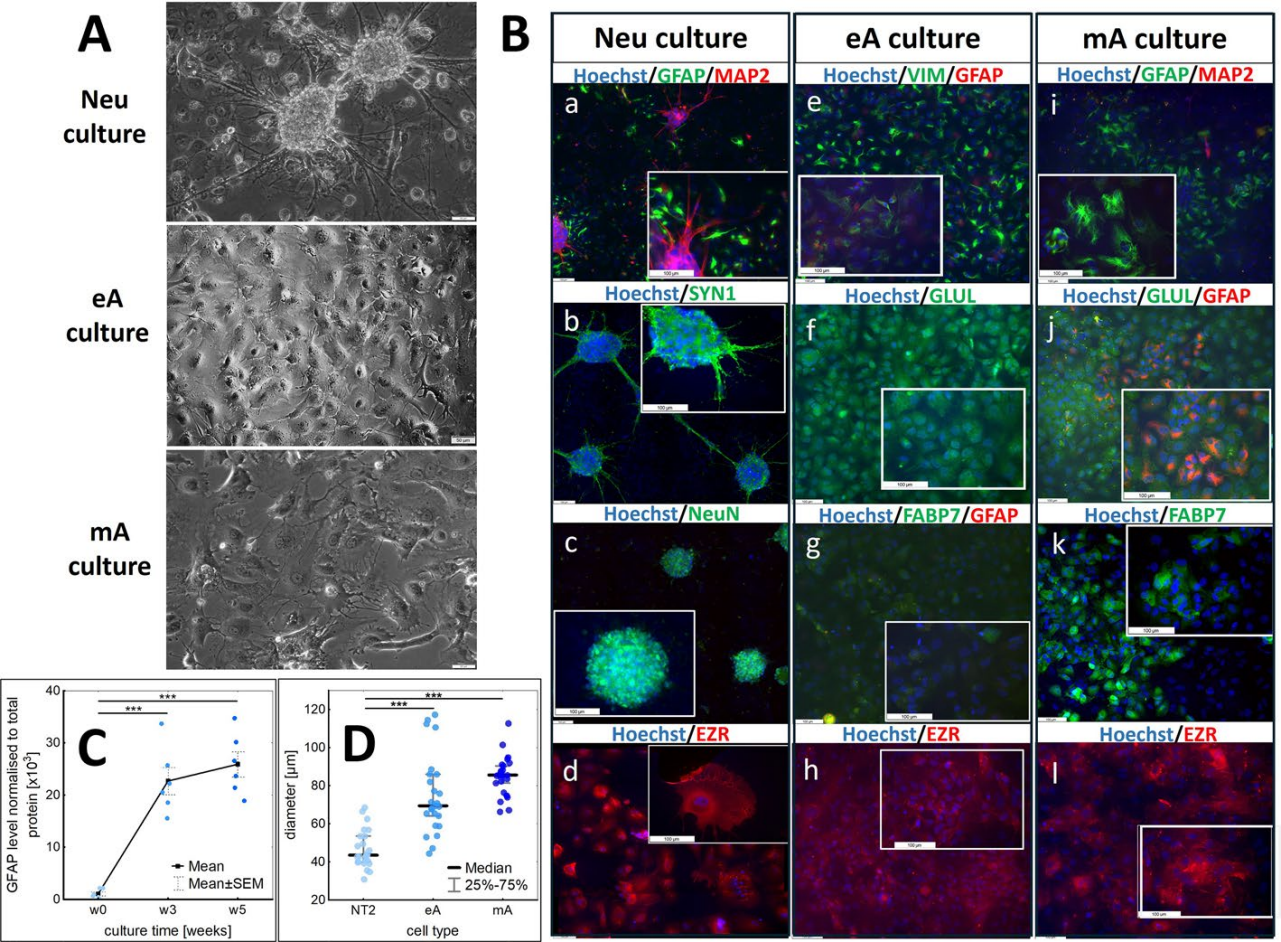
**A** Representative morphological features of Neu (neuron-enriched), eA (early astrocytes), and mA (mature astrocytes) cultures. Scale bars always indicate 50 µm. **B** Immunocytochemical staining for neuronal and astrocytic markers. Each tile scan is composed of 20 images. Supplementary Fig. S5A shows the images without zoomed inserts, while Supplementary Fig. S5B presents additional images of neuronal staining in mA cultures. Scale bars always indicate 100 µm. **C** Time-dependent changes in the GFAP level upon astrocyte maturation monitored using Western blot with densitometry signals for GFAP normalized to the total protein load per well, as determined by ruthenium staining, with available Supplementary Data F1B and Supplementary Fig. S4. w0 denotes the starting point of maturation after the completion of retinoic acid treatment. **D** The distribution of cell diameters for eA and mA relative to undifferentiated NT2 cells, with *n* = 25, was calculated using ImageJ software, assuming a circular cell shape. Owing to variance inequality, the Kruskal–Wallis test was used for statistical analysis

The progress of RA-induced NT2 differentiation was monitored, and the alterations in cell morphology are shown in Supplementary Fig. S1. Undifferentiated NT2 cells were characterized by an irregular shape of the cell body (Supplementary Fig. S1, day 1), a moderate rate of cell division (doubling time of ~ 3.2 days), and a tendency toward growing in monolayer. During the first week of differentiation with RA (without U), the cells continued to divide and form a dense, multilayered culture. Toward the end of the second week and at the beginning of the third week of culturing, round cell clusters/aggregates of neuronal precursors became visible above a layer of adherent cells (Supplementary Fig. S1, day 15). Accutase-driven detachment of all the cells after 4 weeks following RA treatment allowed the neuronal precursors in the clusters to be separated from each other, and because they were less adherent than the other cells, they could have been separated mechanically the next day. These less adherent cells constituted about 30% of the original cells used for differentiation. The course of maturation of neurons and astrocytes is shown in Supplementary Fig. S2 and S3, respectively. Representative pictures of Neu, eA, and mA cells are presented in Fig. 1A.

Determination of the purity of the final neuronal culture is difficult as, during maturation, neurons form aggregates of cell bodies with long cellular protrusions. Although it is possible to obtain a nearly pure neuronal culture (with about 90% of neurons) on a microscope slide, if a higher number of cells is needed and maturation is conducted in the culturing flask, an enriched culture is usually obtained.

Figure 1 presents the results obtained for a neuron-enriched culture of 60% purity (60% of neurons to 40% of astrocytes) after 3 weeks of maturation following RA treatment as well as for a pure cell culture of early astrocytes (> 98%)—after 3 weeks of maturation, and for mature astrocytes (95% of astrocytes to 5% of neurons)—after 6 weeks of maturation. By “pure” we mean containing no more than 5% of other cells.

### Cell phenotype and general marker characteristics

The presence of several protein markers specific to neurons and astrocytes was examined in the differentiated cells using immunocytochemistry staining, as depicted in Fig. 1B.

Only the cells displaying a neuron-like morphology showed positive staining for the following, widely recognized neuronal markers: MAP2 (microtubule-associated protein 2), SYN1 (synapsin 1), and NeuN (neuronal nuclei antigen also known as RNA binding protein fox-1 homolog 3, RBFOX3) (Fig. 1B a–c). The staining specificity was confirmed on the basis of the proper subcellular localization of the signal. MAP2 and SYN1 were detected in all neurons in the neuron-enriched culture, while NeuN presence was confirmed in the majority of neuronal nuclei within this culture. Localization of NeuN outside the nucleus, specifically in the perinuclear cytoplasm, has also been reported [15]. As Neu culture contained astrocytes, staining for astrocyte markers such as GFAP (glial fibrillary acidic protein; Fig. 1B a) was also positive in a cell subset, as it was for EZR (ezrin Fig. 1B d) in the vast majority of astrocytes in the culture.

Immunostaining of early astrocytes eA (Fig. 1B e–h) revealed the presence of VIM (vimentin) and GLUL/GS (glutamine synthetase) in all cells, although some undifferentiated NT2 cells were also positive for GLUL and VIM (Supplementary Fig. S5D). GFAP and FABP7 (fatty acid-binding protein 7) were absent in undifferentiated NT2 cells (Supplementary Fig. S5D), whereas in eA cultures, only a very small number of cells showed a weak signal for GFAP and FABP7 (Fig. 1B e, g). In eA culture, cells showed relatively strong EZR staining, but we also found EZR-positive cells in a relatively large subset of NT2 cells (60%).

To summarize, the presence of the neuronal markers (MAP2, SYN1, and NeuN) was confirmed by immunocytochemistry in all cells exhibiting a characteristic neuronal morphology in the Neu culture, as well as in the few neurons present in astrocyte cultures. The morphology of neurons and astrocytes in Neu culture is presented in video “Neu_culture_200um.mov”. The video monitored cells in Neu culture over 72 h during the third week of maturation, revealing neurons' tendency to form spheres and the active participation of astrocytes in this process. The mA culture contained only a small number of neurons, not exceeding 5%, whereas the eA culture had an even lower number of neurons, accounting no more than 2% of the total cell count.

In mature astrocyte, mA, cultures (Fig. 1B i–l), all the cells were VIM- and GLUL/GS-positive (Supplementary Fig. S5B and Fig. 1B j, respectively), but only approximately 40% of the cells were clearly GFAP-positive (Fig. 1B i, j). GFAP-immunoreactive cells were characterized by a more fibrillar shape compared with the GFAP-negative cells within the same culture, and they were located in areas of higher density. Additionally, 50% of mA cells were strongly positive for FABP7 (Fig. 1B k). All cells clearly stained with GFAP were also strongly positive for FABP7; however, there were also cells stained with FABP7 antibodies but negative for GFAP. GFAP-stained cells were often found close to neurons in neuron-enriched cultures, Neu. Cells with FABP7 and GFAP colocalization, as well as cells positive only for FABP7, were also identified in Neu cultures, albeit in smaller numbers than in the mA cultures. The level of GFAP protein increased gradually with astrocyte maturation after RA treatment, as demonstrated by Western blot in Fig. 1C. mA cells exhibited strong staining for EZR. Astroglial cells are the only ezrin-expressing cells in the normal, adult mammalian CNS, but during brain development, EZR is present also in axon terminals of select neuronal subtypes [16]. In astrocytes, EZR is localized in the peripheral astrocyte processes (PAPs), which constitute 70–80% of the astrocyte cell surface [16]; indeed, such cellular localization was seen in Neu, eA, and mA, but not in NT2 cells, where the signal was detected in the form of dots (Fig. 1B d, h, l; Supplementary Fig. S5D for comparison).

While neurons exhibited a distinct, easily recognizable morphology, astrocytes displayed a diverse range of shapes and sizes. The surface area of undifferentiated NT2 cells, as well as of early (eA) and mature (mA) astrocytes, was measured. Early astrocytes (eA) are larger than NT2 cells, with diameters ranging from 50 to 120 µm and a median diameter of approximately 70 µm (Fig. 1D). Mature astrocytes (mA) exhibit less size heterogeneity and are predominantly large, flattened cells with a median diameter of about 85 µm (Fig. 1D).

According to Sandhu et al. [8], 3-week-old astrocytes typically have a diameter of about 95 µm, which corresponds well to 6-week-old mA cells in our study, whose diameters ranged from 75 to 115 µm (with a median diameter of approximately 85 µm). The size of eA cells varied, with an average diameter noticeably smaller than that of mA cells. In line with prior findings, eA cultures exhibited a certain proportion of fibrous/stellate astrocytes growing atop the protoplasmic astrocytes, while the 6-week-old mA cultures were predominantly composed of flat, large, nondividing protoplasmic astrocytes. The morphology of mature astrocytes in mA culture is depicted in Supplementary Fig S5C. Additionally, the video “mA_culture_100um.mov” provides a closer look at morphological details. This video monitored astrocytes in mA culture for 72 h during the sixth week of maturation, demonstrating their viability and motility. There it is also possible to observe motile astrocyte protrusions.

Astrocytes in the mA culture were quiescent and did not exhibit any proliferative activity. Moreover, mA cells actively participate in calcium wave propagation following ATP stimulation, demonstrating their functionality, as shown in the video “mA_calcium_signaling.mov”.

The major advantage of immunocytochemical staining is its ability to precisely localize and visualize specific proteins within cells, thereby facilitating the study of protein distribution in heterogeneous cell cultures. However, a general question arises regarding how many and which markers should be utilized for the reliable determination of cell identity.

A proteomic approach provides the opportunity for a much broader exploration of the protein profile of cells and for the understanding of their true phenotype. Fractionation of protein samples obtained by cell lysis allowed us to identify large sets of proteins in each studied cell type (Table 1).

**Table 1:** Proteomic profiling of the studied cell types

Cell type	No. of biological replicates	Mean no. of proteins in sample within the group (at least 1 razor + unique peptide)	Mean no. of proteins in sample within the group (copy number analysis)	No. of nonredundant proteins in group (at least 1 razor + unique peptide)	No. of nonredundant proteins in group (copy number analysis)
Neu	6	6844 ± 581	6730 ± 609	8117	8037
eA	5	5897 ± 357	5756 ± 345	7306	7175
mA	6	6602 ± 258	6462 ± 268	7852	7740
NT2	8	6027 ± 896	5891 ± 919	8185	8095

After removing contaminants, a total of 9113 nonredundant proteins were identified in the experiment.

The relative protein abundance within each group was estimated by taking the base_10_ logarithm of the mean copy numbers within the group. The mean log_10_ copy number for all proteins in this study was 4.3, consistent with previous reports on experiments with a high number of identified proteins [17]. However, the range of log_10_ protein copy numbers spanned from 0.5 to 8.3, indicating that the actual number of protein copies per cell varied from approximately 3 to 100 million.

**Fig. 2:**
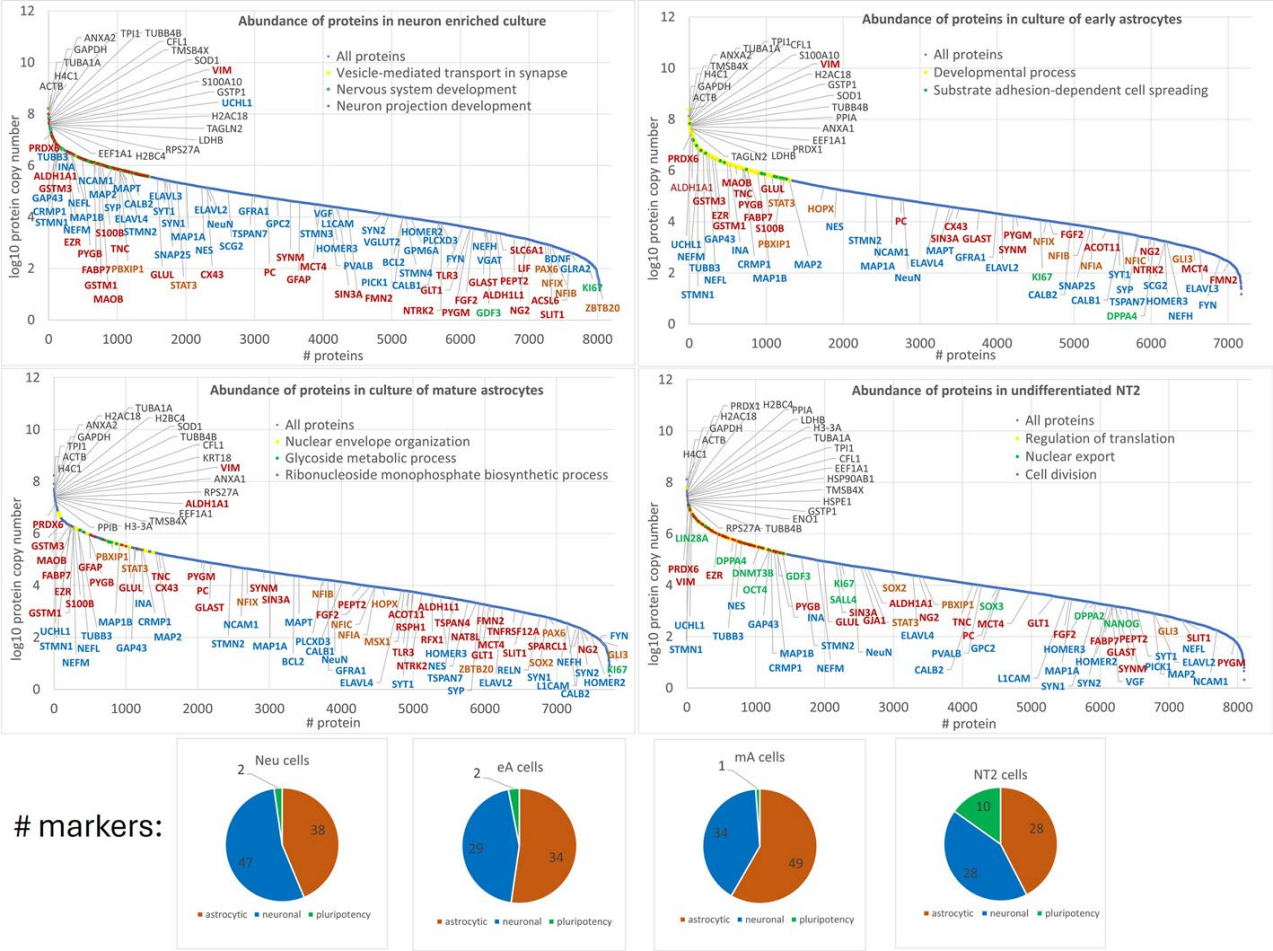
Protein abundance in each cell type represented by protein copy numbers. The 20 most abundant proteins within the group are highlighted in black above the curve. Neuronal markers, astrocytic markers, pro-astrocytic transcription factors, and markers of pluripotent cells are marked in blue, red, orange, and green, respectively. Additionally, the enriched biological processes associated with the most abundant proteins in the first quartile (Q1) obtained in g:Prolifer (https://biit.cs.ut.ee/gprofiler/gost) are depicted in the figure. Pie plots depict the numbers of markers listed in Table 2 identified in the given cell types

The 20 most abundant proteins within each group are listed in Fig. 2. Additionally, 48 markers for neurons, 53 for astrocytes, and 10 for pluripotent cells, identified in an exhaustive literature survey, are indicated in Fig. 2. For proteins ranking in the first quartile for abundance, a Gene Ontology (GO) enrichment analysis was performed and Fig. 2 illustrates the biological processes that distinguish the four cell groups studied.

**Table 2:** The identified astrocytic, neuronal, and pluripotency markers depicted in Fig. 2

Uniprot ID	Gene name(s)	Mean no. of razor + unique peptides				Log_10_ mean protein copy number				Statistical significance in pair comparison					
		Neu	eA	mA	NT2	Neu	eA	mA	NT2	Neu/NT2	eA/NT2	mA/NT2	Neu/mA	eA/mA	Neu/eA
**Neuronal markers**															
***Synaptic proteins***										+/* means p adj < 0.05					
P23416	GLRA2	0.3	—	—	—	2.6	—	—	—						
P51674	GPM6A	2.2	—	—	—	3.9	—	—	—						
Q9H598	SLC32A1/VGAT	1.3	—	—	—	3.5	—	—	—						
Q9P2U8	SLC17A6/VGLUT2	2.3	—	—	—	4.1	—	—	—						
P32004	L1CAM	6.0	—	0.2	2.4	4.4	—	2.4	3.5	+					
P17600	SYN1	20.0	0.2	0.7	1.5	5.4	—	2.8	3.3	*		*	*		
Q92777	SYN2	2.3	—	0.3	1.0	4.1	—	2.3	3.2						
Q9NSB8	HOMER2	0.8	—	0.5	0.6	3.9	—	2.1	3.2						
P13521	SCG2	11.8	0.4	—	—	5.0	3.2	—	—						
P60880	SNAP25	8.5	0.8	—	—	5.4	3.8	—	—						+
P08247	SYP	4.3	0.2	0.5	—	5.9	3.6	3.3	—						
Q9NSC5	HOMER3	2.5	0.6	1.2	0.8	4.4	3.2	3.6	3.4						
P13591	NCAM1	25.0	10.2	10.0	0.4	6.1	4.9	4.7	2.2				*		*
P21579	SYT1	10.7	0.6	2.7	0.4	5.4	3.6	3.7	3.0				*		*
P17677	GAP43	15.8	8.6	11.2	6.4	6.8	6.5	5.4	5.4	+	+		+	+	
Q8N158	GPC2	6.8	—	—	2.4	4.7	—	—	3.9	*					
P41732	TSPAN7	1.5	0.4	0.8	—	4.8	3.4	3.5	—				+		
***Cytoskeletal proteins***															
Q9NZ72	STMN3	1.2	—	—	—	4.5	—	—	—						
Q9H169	STMN4	0.3	—	—	—	4.0	—	—	—						
P16949	STMN1	6.3	5.0	4.3	6.1	6.9	6.9	6.3	6.9			+	+	+	
Q93045	STMN2	2.2	0.8	0.8	0.9	5.8	5.1	4.7	5.1	*			*		
P10636	MAPT/TAU	8.3	2.2	3.0	—	5.8	4.6	4.3	—				+	+	+
P78559	MAP1A	50.7	37.0	26.2	3.0	5.3	5.0	4.5	3.5				+	+	+
P46821	MAP1B	97.7	70.4	62.3	58.3	6.4	5.9	5.6	5.3	*	*	*	*	*	*
P11137	MAP2	61.5	44.4	42.3	1.0	5.9	5.6	5.2	2.9	*	*	*	*	*	*
Q13509	TUBB3	15.5	13.4	12.3	11.1	7.1	6.7	6.2	5.7	+	+	+	+	+	+
P12036	NEFH	0.8	0.6	0.2	—	3.5	3.2	2.4	—						
P07197	NEFM	47.3	41.6	41.2	3.4	6.6	6.8	6.2	5.0	*	*	*	*	*	*
P07196	NEFL	29.2	23.8	24.0	0.1	6.5	6.6	6.2	2.6				+	+	
Q16352	INA	30.3	19.2	12.3	13.1	6.8	6.2	5.4	5.2	*	*		*	*	*
P48681	NES	27.3	26.2	4.7	44.8	5.2	5.3	3.4	5.6	*	*	*	*	*	
***RNA binding proteins***															
Q14576	ELAVL3	7.7	0.2	—	—	5.4	2.9	—	—						
Q12926	ELAVL2	4.0	1.6	1.0	0.8	5.1	4.3	3.1	2.9						*
P26378	ELAVL4	14.2	2.6	2.2	1.4	5.8	4.6	3.8	4.4	*			*	*	*
O43251	RBFOX3/NeuN	5.8	3.8	3.0	4.1	5.1	4.9	4.2	4.7	+		+	+	+	
***Regulatory proteins***															
P23560	BDNF	0.2	—	—	—	2.6	—	—	—						
O15240	VGF	6.0	—	—	0.6	4.5	—	—	3.2						
P56159	GFRA1	6.8	2.4	4.8	—	4.8	4.3	4.2	—						
P06241	FYN	1.3	0.2	0.3	—	3.8	2.6	1.9	—						
Q14194	CRMP1	34.3	22.8	15.7	17.1	6.8	6.1	5.4	5.3	*	*		*	*	*
P10415	BCL2	0.5	—	1.5	—	4.0	—	4.4	—						
Q63HM9	PLCXD3	1.7	—	0.5	—	3.8	—	4.2	—						
P78509	RELN	—	—	2.3	—	—	—	2.8	—						
P09936	UCHL1	13.8	14.4	11.2	10.8	7.6	7.5	7.2	6.8	+	+	+	+	+	
P20472	PVALB	0.8	—	—	0.8	4.4	—	—	4.0						
P05937	CALB1	1.2	0.4	2.5	—	3.8	3.6	4.1	—						
P22676	CALB2	10.5	0.2	0.3	0.8	5.7	3.9	2.5	4.0						
Q9NRD5	PICK1	2.7	0.2	—	1.1	4.0	3.1	—	3.1						

Uniprot ID	Gene name(s)	Mean no. of razor + unique peptides				Log_10_ mean protein copy number				Statistical significance in pair comparison					
		Neu	eA	mA	NT2	Neu	eA	mA	NT2	Neu/NT2	eA/NT2	mA/NT2	Neu/mA	eA/mA	Neu/eA
**Astrocytic markers**															
***Transcription factors***										+/* means p adj < 0.05					
P28360	MSX1	—	—	1.3	—	—	—	3.8	—						
Q33E94	RFX4	—	—	0.5	—	—	—	2.7	—						
Q9BPY8	HOPX	—	1.2	0.2	—	—	5.3	3.9	—						
P10071	GLI3	—	0.6	0.2	0.8	—	3.1	1.8	2.8		+				
Q96AQ6	PBXIP1	19.5	14.0	17.8	6.5	5.8	5.7	5.7	4.3	+	+	+			
Q12857	NFIA	—	0.8	1.0	—	—	3.8	3.9	—						
O00712	NFIB	0.2	1.0	1.7	—	2.9	3.8	4.2	—					+	
P08651	NFIC	—	0.8	1.7	—	—	3.4	3.9	—						
Q14938	NFIX	0.3	1.4	4.2	—	2.9	4.0	4.6	—					+	
P26367	PAX6	0.3	—	0.7	—	3.0	—	2.8	—						
Q9HC78	ZBTB20	0.2	—	0.8	—	2.0	—	3.3	—						
P48431	SOX2	—	—	0.3	2.1	—	—	2.5	4.5						
P40763	STAT3	18.5	18.0	17.5	9.1	5.3	5.7	5.4	4.7	*	*	*		*	*
***Regulatory proteins***															
P09038	FGF2	1.0	0.6	1.5	0.9	3.5	4.0	4.0	3.5						
PVALBP24821	TNC	57.7	57.6	30.3	6.1	5.8	6.1	5.2	4.0				+	+	+
O75093	SLIT1	0.7	—	0.7	0.3	2.9	—	3.0	2.6						
Q14515	SPARCL1	—	—	0.7	—	—	—	2.4	—						
Q9NP84	TNFRSF12A	—	—	0.3	—	—	—	3.0	—						
O15455	TLR3	2.3	—	4.0	—	3.6	—	3.8	—						
P04271	S100B	2.0	1.8	2.3	—	6.0	5.9	6.2	—					+	+
O15540	FABP7	7.7	7.8	8.3	0.4	6.1	6.0	6.3	3.2					+	+
P17302	GJA1/CX43	5.5	2.2	8.5	2.9	5.1	4.5	5.2	4.7			*		*	*
Q16620	NTRK2	1.2	0.6	2.2	—	3.8	3.4	3.7	—						
P15018	LIF	0.7	—	—	—	3.1	—	—	—						
Q96ST3	SIN3A	11.0	10.4	16.3	24.4	4.4	4.6	4.5	4.8	*	*	*			
P35442	THBS2	1.3	0.6	0.2	0.1	3.4	2.9	—	2.1						+
Q6UVK1	CSPG4/NG2	2.8	2.0	1.0	9.8	3.1	3.4	2.3	4.4			*	*	*	
***Cytoskeletal proteins***															
P14136	GFAP	3.5	—	22.3	—	4.6	—	6.0	—					+	
P08670	VIM	49.0	40.6	41.2	31.3	7.7	7.8	7.4	6.9	*	*	*	*	*	
P15311	EZR	28.3	22.4	22.5	18.5	6.5	6.5	6.3	6.0	+	+	+			
O15061	SYNM	15.8	8.0	18.0	1.1	4.6	4.2	4.5	3.2						*
Q9NZ56	FMN2	6.8	0.2	2.7	—	4.3	2.4	3.2	—				+		
***Transporters/membrane proteins***															
P43004	SLC1A2/GLT-1	1.2	—	0.2	0.1	3.6	—	3.2	3.6						
P43003	SLC1A3/GLAST	0.8	2.0	4.2	0.5	3.5	4.4	4.9	3.2				+	+	+
O15427	SLC16A3/MCT4	3.2	0.2	1.7	1.0	4.5	3.0	3.2	3.7				+		
P30531	SLC6A1/GAT-1	0.5	—	—	—	3.0	—	—	—						
Q16348	SLC15A2/PPT2	0.2	—	2.8	0.1	3.2	—	4.0	3.1						
P50993	ATP1A2	—	—	0.2	3.3	—	—	1.6	3.8						
O14817	TSPAN4	—	—	0.2	—	—	—	3.5	—						
Q8WYR4	RSPH1	—	0.2	1.0	—	—	2.8	3.8	—						
***Metabolic proteins***															
Q8N9F0	NAT8L	—	—	1.0	—	—	—	3.3	—						
Q8WXI4	ACOT11	—	1.6	1.3	—	—	3.7	3.8	—						
O75891	ALDH1L1	1.0	—	0.3	—	3.4	—	3.5	—						
P27338	MAOB	21.7	20.4	21.8	—	6.0	6.2	6.3	—				*	*	
P00352	ALDH1A1	28.8	38.6	37.3	2.5	6.8	7.3	7.4	4.7	*	*	*	*		*
P11217	PYGM	2.5	3.2	17.8	0.3	3.6	4.2	4.9	1.8				+	+	
P11498	PC	14.0	11.6	18.5	5.3	4.8	4.9	4.9	3.9	*	*	*			
P30041	PRDX6	21.5	21.4	20.7	19.5	7.3	7.5	7.2	7.1	+	+	+		+	+
P15104	GLUL/GS	8.2	10.0	6.8	5.8	5.5	5.8	5.5	4.9	+	+	+		+	+
P09488	GSTM1	10.3	13.2	12.3	9.0	6.1	6.3	6.2	5.5	+	+	+	+		+
P11216	PYGB	46.0	38.6	34.7	25.5	6.3	6.1	6.0	5.3	+	+	+	+		
P21266	GSTM3	19.8	17.8	14.7	6.1	6.8	6.7	6.5	5.3	*	*	*	*	*	
Q9UKU0	ACSL6	0.7	—	—	—	2.6	—	—	—						

Uniprot ID	Gene name(s)	Mean no. of razor + unique peptides				Log_10_ mean protein copy number				Statistical significance in pair comparison					
		Neu	eA	mA	NT2	Neu	eA	mA	NT2	Neu/NT2	eA/NT2	mA/NT2	Neu/mA	eA/mA	Neu/eA
**Pluripotency markers**										+/* means *p*_**adj**_** < 0.05**					
Q9H9Z2	LIN28A	—	—	—	15.8	—	—	—	6.9						
Q9UBC3	DNMT3B	—	—	—	22.6	—	—	—	5.5						
Q01860	POU5F1/OCT4	—	—	—	7.8	—	—	—	5.4						
Q9NR23	GDF3	0.2	—	—	6.6	3.4	—	—	5.3						
Q9UJQ4	SALL4	—	—	—	15.1	—	—	—	4.8						
P41225	SOX3	—	—	—	1.0	—	—	—	3.9						
Q7Z7J5	DPPA2	—	—	—	0.6	—	—	—	3.3						
Q9H9S0	NANOG	—	—	—	0.6	—	—	—	3.1						
Q7L190	DPPA4	—	0.2	—	8.5	—	3.3	—	5.8						
Q9BYG3	KI67	6.3	5.4	5.5	11.8	5.0	5.0	4.8	5.6	+	+	+		+	

+ parametric test (ANOVA with post hoc Tukey test), * nonparametric test (Kruskal–Wallis and Mann–Whitney/Wilcoxon tests), quantitative analysis was conducted if values were present for at least 60% of the samples in a group (see Supplementary Figs. S6–S9 for details). Full protein names, along with related references and information about species, are listed in Supplementary Table S2.

### Neuronal protein markers

Proteomic analysis revealed the presence of ~45 previously reported neuronal markers in the neuron-enriched cultures, Neu (Fig. 2; Table 2). UCHL1 was the most abundant marker, while BDNF, GLRA2, GPM6A, STMN3, STMN4, VGAT, and VGLUT2 were identified as the most specific markers for neurons. BCL2 and PLCXD3 were found in Neu and mA cultures. Several neuronal markers, including L1CAM, SYN1, and SYN2, were found at a significantly higher level in Neu than in mA or NT2 cultures, whereas ELAVL3, SCG2, and SNAP25 were present at a high level in Neu and at a much lower level in eA cultures.

The quantitative analysis of protein copy numbers across the cell types studied confirmed a high number of many widely recognized neuronal markers in the Neu culture. Among them were synaptic proteins (NCAM1, SYN1, SNAP25, SYT1, GAP43, GPC2, and TSPAN7; Supplementary Fig. S6 a–g), cytoskeletal proteins (MAP1A, MAP1B, MAP2, and TUBB3; Supplementary Fig. S6 h–k), and RNA-binding proteins, such as ELAVL2, ELAVL4, and RBOX2 (Supplementary Fig. S6 l–n) as well as important regulatory proteins, such as GFRA1, CRMP1, UHL1, and CALB1 (Supplementary Fig. S6 o–q, s).

In Neu cells, apart from proteins involved in the regulation of microtubule structure and dynamics, there were proteins engaged in the transport, storage, and release of neurotransmitters (including glutamate and GABA), neurotrophins, as well as proteins with roles in neuron development, synapse formation, and plasticity (VGLUT2, VGAT, GLRA2, BDNF, SCG2, GPM6A, HOMER2, and HOMER3).

Among the proteins strongly upregulated in the Neu culture were members of the embryonic lethal abnormal vision (ELAVL) protein family, also known as the human antigen D (HuD), such as ELAVL2, ELAVL3, and ELAVL4, which play a crucial role in the posttranscriptional regulation of expression of the genes important for learning and memory in mammals. The above-mentioned proteins target genes encoding neurotrophic factors, e.g., BDNF, which we detected only in Neu cells, and growth-associated protein-43 (GAP43, neuromodulin) [18]. Clearly, the highest level of GAP43 was found in the Neu culture (Fig. 2; Table 2). Another RNA-binding protein strongly upregulated in Neu cells was NeuN/RBFOX3. The presence of RBFOX3 was confirmed by immunostaining (Fig. 1B c). In LC–MS/MS analysis, the protein was identified as a protein group containing RBFOX1 and RBFOX2, with its highest level detected in the Neu culture. Sequence identity between RBFOX3 and RBFOX1 is ~78% and that between RBFOX3 and RBFOX2 is ~72%. RBFOX proteins regulate the splicing program, which plays a crucial role in the structural and functional maturation of postmitotic neurons [19].

CRMP1 is involved in dendritic development and spine maturation, and Tyr504 phosphorylation of CRMP1 by Fyn is an essential step of Sema3A-regulated dendritic development of cortical pyramidal neurons [20]. The expression of Fyn is high in neurons but low in nonreactive astrocytes [21]. In our study, Fyn was present at a very high level in Neu cells. Its level in eA and mA cells was moderate and low, respectively, and the protein was absent from NT2 cells (Fig. 2; Table 2). Fyn-mediated signaling pathways are involved in neuronal differentiation and plasticity [21]. CRMP1 is also involved in reelin-regulated neuronal cell migration. Surprisingly, a low level of RELN known as a neuronal marker, was detected only in the mA cultures, which contained a very small number of neurons (Fig. 2; Table 2). It has been shown that a developmental subset of astrocytes expresses RELN along with SLIT1 and PAX6 [22, 23]. In our study, we found SLIT1 and PAX6 present at very low levels in the Neu and mA cultures (Fig. 2; Table 2).

L1CAM protein was found at a high level in Neu and at a lower level in mA and NT2, and was completely absent from eA cells (Fig. 2; Table 2). The protein is required for cell adhesion and migration and also for axonal and dendritic arborization and action potential generation [24]. On the other hand, the complex of receptor GFRα1 (Supplementary Fig. S6o; Neu > eA, mA) along with its ligand GDNF (glial cell line-derived neurotrophic factor) promotes the development of hippocampal dendritic arbors and spines via NCAM (Supplementary Fig. S6a; Neu > eA, mA) [25]. The GDNF/GFRa1 complex is essential for the plasticity of hippocampal adult circuits [26]. Conversely, in the mouse cortex, it promotes neuronal differentiation by inhibiting the self-renewal capacity of neural precursors induced by fibroblast growth factor 2 (FGF2) [27]. Glypican-2 (GPC2; Supplementary Fig. S6f) also binds FGF2 and its receptor, impeding neural progenitor cell proliferation by antagonizing the mitotic effect of FGF2 [28]. In our study, FGF2 was found at a lower level in Neu cells compared with those in eA and mA cells (Fig. 2; Table 2). This observation is in line with previous findings indicating that the activation of FGF signaling suppresses neurogenesis and promotes astrocytogenesis during mammalian neocortex development [29].

In the Neu culture, a substantial concentration of the neurosecretory protein VGF (also known as secretogranin VII) was observed (Fig. 2, Table 2). VGF is expressed by GABAergic and glutamatergic neurons. The expression of VGF mRNA is significantly enhanced by neurotrophic factors, including BDNF [30]. VGF and its derived peptides play pivotal roles in regulating energy balance, synaptogenesis, neurogenesis, as well as in learning and memory, both in the developmental stages and throughout adulthood [31].

Another regulatory protein found exclusively in Neu and mA cultures was BCL2 (Fig. 2; Table 2), which protects neurons from apoptosis during the developmental stage and is also involved in the survival and maintenance of mature neurons. An elevated electrical activity of neurons results in a lasting increase in BCL2 expression. Neurons exhibiting spontaneous activity primarily demonstrate BCL2 expression without the presence of BAX [32].

The nature of the neurons can be more accurately delineated by examining their receptor profiles and by identifying specific proteins characteristic of the specific neuron type. Exclusively in Neu samples, subunits of GABAA and GABAB receptors, as well as subunits of NMDA and AMPA receptors, were identified. Additionally, muscarinic acetylcholine receptors M2 were detected in Neu and mA (Neu > mA) (Supplementary Table S3, receptors). Astrocytic muscarinic acetylcholine receptors are known to accelerate neuronal development [33]. Cortical human GABAergic interneurons can be distinguished based on the expression of calcium-binding proteins, such as PVALB (parvalbumin), CALB1 (calbindin), and CALB2 (calretinin). These proteins modulate neuronal activity. In the Neu culture, we observed a high abundance of CALB2 (Table 2).

The functional maturation of glutamatergic synapses was confirmed by the abundant presence of the scaffold protein TSPAN7, recognized as a pivotal molecule in both the functional and morphological maturation of dendritic spines. Collaborating with PICK1, TSPAN7 facilitates the internalization of AMPA receptors, a process critical for excitatory synapse development [34]. Both TSPAN7 and PICK1 exhibited their clearly highest expression levels in Neu cells (Table 2). TSPAN7 enhances the surface expression of dopamine D2 receptors [35]. Furthermore, we observed THBS2/TSP2 (thrombospondin 2), an astrocytic protein known to promote neurite outgrowth, at its highest level in the Neu cells (Neu > eA > NT2, Table 2).

### Intermediate filaments

Intermediate filaments (IFs) are important effector proteins that clearly differentiate brain cell types at various stages of development. We identified 67 proteins belonging to the Gene Ontology term GO:0045111, intermediate filament cytoskeleton, cellular component (Supplementary Table S2, IFs). In the Neu culture, a high amount of neurofilaments such as NFL, NFM, NFH, and INA (Supplementary Fig. S7A a–d) was noticed, but, surprisingly, an even higher level of these proteins was found in eA cells. GFAP, important astrocytic marker, was identified only in mA and Neu cells, with a much higher level in the mA culture (Supplementary Fig. S7A e). Results regarding other IFs are shown in detail in Supplementary Table S2 and Supplementary Fig. S8.

### Astrocytic protein markers

Table 2 lists approximately 50 astrocytic markers reported in the literature. The majority of them were present in the mA culture, whereas a smaller subset was identified in eA cells (Fig. 2). Proteomic analysis confirmed the presence of the most commonly accepted markers for astrocytes, namely GFAP and ALDH1L1 in mA and Neu cultures. These markers were absent from eA and NT2 cultures. Several well-confirmed, specific astrocytic markers, such as VIM, PRDX6, GSTM3, GSTM1, and PYGB, were present in mA and eA cells in very high copy numbers (Fig. 2). We also found EZR, which is predominantly expressed in astrocytes [36], at a high level in Neu and eA and at a slightly lower level in mA (Fig. 1B d, h, l and Supplementary Fig. S7 g). The most specific proteins for mA were NAT8L, TNFRSF12A, TSPAN4, and RSPH1. Additionally, TRL3 was present in mA and Neu cultures (Table 2).

### The profile of transcription factors and transcriptional co-activators in NT2-derived astrocytes

We identified several transcription factors and transcriptional co-activators, which underscore the complexity of astrocyte-specific markers (Supplementary Table S4). The most specific for mA were transcription factors MSX1 and RFX. RFX4 was exclusively found in mA, indicating its potential as a unique marker for this astrocyte subtype. In mouse hippocampus, MSX1 was found mainly in astrocytes [37]. According to the literature [38], RFX4 together with GLI3 and PBXIP1 can be considered astrocyte-specific markers. In this study, GLI3 displayed its highest expression levels in eA, followed by mA, and it was absent from Neu. At the same time, PBXIP1 showed similar levels in mA, eA, and Neu, all higher than in NT2. Interestingly, another member of the regulatory factor X family, RFX2, was observed at significantly higher levels in mA than in the other cell types studied.

Other important transcription factors that drive astrocyte differentiation were identified in mA, specifically the following isoforms of nuclear factor I: NFIA, NFIB, NFIC, NFIX, and also ZBTB20, HOPX, and PAX6. In eA, NFIA, NFIB, NFIC, NFIX, and HOPX were observed, while NFIB, NFIX, ZBTB20, and PAX6 were present in Neu. Several of them were detected at a very low level, which was expected in the case of transcription factors (Supplementary Table S4).

NFIA, which functions as a molecular switch of glial competency, was detected only in mA and eA cells (mA > eA). Even transient expression of NFIA is enough to stimulate the ability of human neural stem cells to become glial cells. Interestingly, astrocytes induced by NFIA facilitate synaptogenesis, demonstrate neuroprotective characteristics, exhibit calcium fluctuations when exposed to suitable stimuli, and integrate into the mature mouse brain. Differentiation involves chromatin remodeling and GFAP promoter demethylation [39]. For NFIA induction, an expression of SOX9 is necessary [40] and the presence of both NFIA and SOX9 is required for astrocyte fating [41]. However, in our proteomic experiment, we detected SOX9 at very low level in mA cells (based on GYDWTLVPMPVR peptide in DIA measurement mode). Nevertheless, considering the proteins identified in eA cells with high confidence, which are not present in NT2 cells, we found SOX9 as an upregulated upstream regulator in eA cells (Supplementary Fig. S12; BH = 0.01, deviation). The synergistic activity between SOX9 and NFIA can be blocked by the presence of SOX3 [42], but we found SOX3, at a very low level, only in NT2 cells. This result is in line with previous reports that showed that SOX3 negatively controls the onset of astrocyte differentiation [42]. However, the expression level of SOX9 gradually declines over the 4 weeks of the NT2-derived astrocyte maturation process, similarly to a decrease in the SOX2 level. SOX2 overexpression in NT2/D1 cells reduces the number of neurons but not astrocytes [43]. It has recently been shown that SOX2 is essential for astrocyte maturation, particularly for the expression of glutamate transporters, SPARCL1, and NTRK2 [44]. As mentioned above, in our study, SOX2 was detected in NT2 cells and—in a low copy number—in mA cells. SPARCL1 and NTRK2 serve as mediators of astrocyte–neuron cross-talk. An astrocyte-secreted protein, SPARCL1/hevin, a positive regulator of synapse formation [45], was detected only in mA cells. NTRK2, which plays a prominent role in astrocyte morphogenesis and contributes to astrocyte regulation of neuronal synapse development [46], was present in the differentiated cells, with slightly higher levels in mA and Neu than in eA. The protein was completely absent from NT2 cells.

All the differentiated cells contained NFIB and NFIX (mA > eA > Neu), but NFIC was present only in mA and eA (mA > eA). NFIB induces functional astrocytes from a human pluripotent stem cell‐derived neural precursor, and NFIB positively feeds back on SOX9 expression [47]. NFIX was found to be a downstream target of NFIB during astrocytic maturation [48], but both NFIC and NFIX are required for the expression of astrocyte markers, including GFAP and SPARCL1 in the later stages of astrocyte differentiation [49]. NFIX also regulates the expression of FABP7 (mA, Neu, eA > > NT2, Supplementary Fig. S7C g) [50]. Another transcription factor promoting astrocytogenesis [51], ZBTB20 (zinc finger- and BTB domain-containing protein 20), was detected at low levels in mA and Neu (mA > Neu). The HOPX transcription factor, detected in mA and Neu cells, was also suggested as a potential astrocyte marker during the differentiation of precursor neural stem cells (NSCs) into astrocytes [52].

### Other important astrocytic markers

In mA cells, markers of astrocyte maturation, including glutamate transporters GLT-1/SLC1A2/EAAT2, and GLAST/SLC1A3/EAAT1, were expressed, with GLAST at a level comparable with that of Neu cells. The eA cells expressed only GLAST. In Neu cells, GLT-1 and GLAST were expressed at similar levels, as a result a higher abundance of GLT-1 compared with that of mA cells. Indeed, it was demonstrated that neuron-free primary astroglial cultures express GLT-1 at very low levels, whereas the presence of neurons in the cultures triggers an increase in GLT-1 expression [53]. GLT-1, the principal glutamate transporter, colocalizes with α2 subunit of Na^+^/K^+^-ATPases in astrocytic processes and with α1 and α3 at axon terminals [54]. GLT-1 expression is strongly induced by FGF2 and other FGF family members, which also enhance astrocyte proliferation and maturation in mouse embryonic stem cell-derived astrocytes [55]. Unexpectedly, astrocytic markers GAT-1/SLC6A1, involved in the reuptake of GABA from the synaptic cleft, as well as protein ACSL6, engaged in fatty acid metabolism, were found only in the Neu culture. This may suggest a higher functionality of astrocytes in the Neu culture. Neurons are known to induce GLT-1 expression in astrocytes through the involvement of the PAX6 transcription factor [56], which was detected in mA and Neu cells in this study. Neuron–astrocyte communication leads to an increase in astrocytic PAX6 levels, thereby stimulating GLT-1, but not GLAST, expression [56]. On the other hand, PAX6 is essential for the differentiation of glutamatergic neurons [57].

The mechanism for glutamine release from astrocytes at synapses remains unclear, yet it likely involves a specific neutral amino acid transporter. In the differentiated cells, we did not detect the astrocyte-specific glutamine transporters SLC38A3/SNAT3 or SLC38A5/SNAT5. However, SLC1A5/ASCT2, whose preferred substrate is glutamine (it is transported bidirectionally), was found in all cell types studied, but it exhibited the highest expression levels in NT2 cells (Supplementary Table S6) [58]. Additionally, SLC38A10, recently shown to bidirectionally transport glutamine in astrocytes [59], was found at higher levels in the differentiated cells in this study (Supplementary Table S6).

In none of the studied cell types did we detect AQP4, which is primarily expressed in the perivascular astrocytic end-feet domains. Endothelial cells promote AQP4 accumulation, so the absence of endothelial cells from our culture explains why the amount of AQP4 was below the detection limit. Similarly, we did not observe Kir4.1/KCNJ10. The reason for this could be a high level of ribosomal protein receptor of activated protein C kinase 1 (RACK1) in all of the studied cell types. The RACK1 protein represses Kir4.1 translation in astrocytes. Ablation of RACK1 in astrocytes results in an increase in the amount of Kir4.1, a larger astrocytic territory volume, longer distal processes, and attenuated recurrent neuronal burst activity [60]. Putative functional coupling of Kir4.1 and AQP4 was suggested for Kir4.1-mediated spatial buffering of K^+^. However, the contribution of Kir4.1 to K^+^ clearance from the extracellular space seems to be smaller than previously assumed, and there is a growing body of evidence that the Na^+^/K^+^-ATPases are mainly responsible for K^+^ clearance [61]. α1 and α3 isoforms of Na^+^/K^+^-ATPase are exclusively neuronal, while α2 is predominantly astrocytic [54]. In the primary culture of rat astrocytes, a shift from the α2 to α1 was reported, but this might have been a culturing effect [62]. We detected α1/ATP1A1 in all cell types at a comparable level, while α2/ATP1A2 was present at a very low level in mA astrocytes (and at a higher level in NT2 cells). α3/ATP1A3 was in higher copy number in Neu, and at a very low level in mA (and a moderate level in NT2 cells).

As shown in Fig. 2, astrocytic proteins were more numerous and more abundant in the mA culture than in the eA and NT2 cultures, but the Neu culture contained a comparable number of astrocytic markers to those of mA cultures, with a slightly different composition of proteins. Without a doubt, the mA culture more extensively reflected astrocytic phenotypes than the eA culture did. Additionally, the Neu culture contained numerous astrocyte-specific proteins at a high level.

### Markers of mature astrocytes

We detected the following, previously used markers for mature astrocytes: S100B, FBXO2, FMN2, NUDT3, S100A1, SYNPO2, PDLIM7, PADI2, TMX2, and NEBL (Supplementary Fig. S7B; Supplementary Table S2). The level of S100B, the most widely recognized marker, was significantly higher in mA cells than in eA or Neu (and it was absent from NT2 cells), but other markers, including FBXO2, FMN2, NUDT3, and CNN1 [63], were more strongly expressed in the Neu culture.

In mature astrocytes of the gray matter, often referred to as protoplasmic ones, high levels of GLT-1/SLC1A2 are expected, while GLAST/SLC1A3 levels should be lower [64]. Conversely, in neural stem cells (NSCs), the opposite relationship appears to be true. In our study, GLT-1 was absent from eA; it was present at lower levels than GLAST in mA, but in the Neu culture it was found at a level comparable to GLAST, which suggests the highest maturity of astrocytes present in the co-culture with neurons.

According to Velloso et al. [64], other proteins are expected to be upregulated in mature astrocytes, such as ADK, CD9, or GLUL. In our studies, the amount of CD9 was slightly higher in mA than in the other cells. At the same time, ADK and GLUL were most abundant in Neu and eA cells, respectively (Supplementary Fig. S9 I–III). A subset of proteins is expected to be downregulated in mature astrocytes relative to immature astrocytes. Indeed, KIF2C, GNL3L, TOP2A, and NES were found at clearly lower levels in mA than elsewhere, while PROM1 level in Neu culture was lowest across the differentiated cells studied (Supplementary Fig. S9 IV–VI; Supplementary Table S2).

Recent single-cell RNA sequencing of early postnatal and adult murine striatal and cortical tissue revealed a common astrocyte maturation signature [65]. We investigated whether the most strongly regulated transcripts also distinguished our experimental groups at the protein level. Many protein counterparts were not identified in our samples; however, among the proteins that were detected, several were uniquely expressed (PHKG1, JOSD2, PTGS1, MFSD6, RNF130, FXYD1, and LYNX1; Supplementary Table S2) or upregulated (PDK2, PYGM, MAOB, S100B, GJA1, RNF13, EPHX2, and SELENBP1; Supplementary Fig. S9 a–d; Supplementary Table S2) in mA cells. Notably, proteins exclusive to mA cells, such as MEGF6, C1QTNF6, SPOCK2, SNN, and RELN, were also upregulated in immature astrocytes [65] (Supplementary Table S2), raising questions about their specificity. Furthermore, FABP7, which was recommended as a marker for immature astrocytes in Lattke’s report, was found at the highest level in mA cells in our study (Supplementary Table S2).

In eA cells, there were fewer proteins uniquely expressed (RHOU, DIXDC1, and OAF; Supplementary Table S2) or upregulated (GLUL, LAP3, ALDOC, GSN, TMOD1, EPS8, and TUBA4A; Supplementary Table S2; Supplementary Fig. S9 III, f–k). Those proteins, according to Lattke’s study, were expected to be at higher levels in mature astrocytes. However, in eA cells, proteins that suggest their immaturity were also found (FAM167A, TCN, MARCKS, and SPARC in Supplementary Fig. S9 ad and CEBPD in Supplementary Table S2). It was shown that transcription factor CEBPD provides an antioxidant effect for astrocytes resistant to intracellular reactive oxygen species [66].

Importantly, in the Neu culture, we found a relatively large group of proteins that were upregulated in mature astrocytes in the single-cell transcriptomic experiment mentioned above. There were proteins exclusively found in Neu cells (ACSL6, APLP1, ATP2B2, CASKIN1, GAS6, GPRC5B, GRIA2, HPCAL4, HS3ST1, KCTD13, NFASC, OLFML1, PPP2R2C, PSD2, RPH3A, SCG3, SLAIN1, SLC6A1, SPOCK1, STMN4, TMEM151A, and WASF3; Supplementary Table S2) or predominantly present in these cells (CLMN, EDIL3, SLC20A1, CADM2, GLT-1/SLC1A2, NAAA, NTRK2, SEZ6L6, CLDN11, SYNGR1, and CNTFR; Supplementary Table S2; Supplementary Fig. S9 o, p, ab). Several other proteins occurred in Neu at a higher level than in the other cell types (CRYAB, GK, ATP1B1, ANK2, BIN1, CLU, GNAO1, HTRA1, PFKP, QDPR, and UPP1; Supplementary Table S2; Supplementary Fig. S9 q–aa). Some of the proteins that were found at a significant level in Neu cells were also present in some other differentiated cells (FGF1 in Supplementary Fig. S9 ac, FAH, PRNP, and SLC27A1 in Fig. S9 l–n, ABCA2, ACSL3 APOE, EZR, LDHB, and NDRG2 in Supplementary Table S2).

However, in Neu cells, we also identified a few proteins expected to be found in immature astrocytes (ABHD17C, CYP1B1, ETV4, EVL, GNG3, OPCML, SLC17A6, and ZDHHC2).

Interestingly, BDNF receptor, NTRK2/TrkB.T1, was statistically significantly upregulated in mA cells (Supplementary Fig. S9 e; Supplementary Table S2). It was recently shown that NTRK2 is highly enriched in astrocytes during the critical period of astrocyte morphological maturation [46].

According to Lattke’s report, the most distinctive finding characterizing immature astrocytes was the upregulation of tenascin C (TNC). In our experiment, TNC was indeed clearly upregulated in eA cells (Table 2). TNC plays a crucial role in sustaining the proliferation and correct morphology of astrocytes in a primary culture [67], and it governs astrocytic reactivity [68].

A surprising discovery in Lattke’s report [65] was the significant upregulation of tubulin polymerization promoting protein (TPPP)/p25 and 2′,3′-cyclic nucleotide 3′-phosphohydrolase (CNP) transcripts in mature astrocytes, a finding that was not addressed in the paper. These proteins are typically recognized as markers for oligodendrocytes and are not anticipated to be present in normal astrocytes. However, they are found in neurons, with TPPP present in both excitatory and inhibitory neurons, and CNP limited to the certain types of excitatory neurons. Additionally, CNP protein is expressed by glial progenitors. The cellular function of TPPP/p25 is associated with microtubule stabilization and cilia formation, while CNP facilitates the process of myelination. We observed a significant increase in TPPP levels and a modest increase in CNP levels in the Neu culture compared with NT2 cells.

Upon considering the proteome profiles and the abundance of specific proteins in the cell cultures studied, it becomes apparent that the eA cell culture contains astrocytes in an immature state. Furthermore, upon examination of the mA and Neu cultures, we noted a substantially greater presence of distinct proteins in the Neu culture compared with that of the mA culture. This result suggests that the neuron-enriched culture (Neu) harbors astrocytes with a more mature morphology compared with those in the mA culture, despite the Neu culture being younger.

### Biochemical pathways characteristic for proteins specifically expressed by different cell types

Proteins present in at least 50% of the samples in each sample group, with the sum of sequenced peptides for a given protein being at least 130% of the number of samples in the group, were subjected to GO enrichment analysis using FunRICH (a *p*-value threshold with a Benjamini–Hochberg correction of less than 0.05 was applied; Supplementary Table S5). Enrichment analysis was performed on a total of 6077, 5282, 5713, and 5482 proteins for Neu, eA, mA, and NT2, respectively.

Multiple biochemical pathways and molecular functions regulated by sets of uniquely and differentially expressed proteins (DEPs) in Neu cells validate the functionality of neurons present in the Neu culture. Neu cells contained proteins indicative of enrichment in the following GO biological processes (BPs): axonogenesis, dendritic spine maintenance, regulation of exocytosis, neurotransmitter secretion, axonal and mitochondrial autophagy, positive regulation of catalytic activity, protein secretion, and the Wnt and TORC1 signaling pathways.

Reactome pathways (RPs) unique to Neu cells encompassed the regulation of insulin secretion by glucagon-like peptide-1 (GLP1), the dopamine neurotransmitter release cycle, the serotonin neurotransmitter release cycle, and the synthesis of phosphoinositides (PIPs) at the early and late endosome membranes.

More information concerning the enrichment analysis of Gene Ontology terms in subsets of proteins uniquely expressed in particular groups can be found in the Supplementary Information.

The main RPs uniquely represented by eA cell proteins were membrane binding and targeting of GAG proteins and TGF-beta receptor signaling that activates SMADs. On the other hand, chaperone-mediated autophagy and the downregulation of TGF-beta receptor signaling were the enriched pathways in mA.

TGF-β is a potent inducer of astrocyte differentiation from radial glia cells (via activation of SMAD2/3 and MAPK signaling pathways) and astrocytic maturation, but it is also an effector of astrocyte function, mediating cross-talk between astrocytes and other cells in the CNS [69]. Neurons at different developmental stages secrete TGF-β that induces different pattern of GFAP expression in astrocytes [70, 71]. On the other hand, murine and human astrocytes induce the formation of excitatory and inhibitory synapses through the secretion of TGF-β. An inflammatory state correlates with increased astrocytic production of TGF-β1, whereas atrophy of astrocytes is associated with a decreased level of astrocytic TGF-β1 [69]. TGF-β2 and TGF-β3 produced by astrocytes also regulate synapse formation and plasticity [72]. Consequently, TGF-β isoforms serve as molecules facilitating two-way communication between neurons and astrocytes. We found TGF-β2 upregulated in differentiated cells (Neu > eA > mA; Supplementary Table S2).

**Fig. 3:**
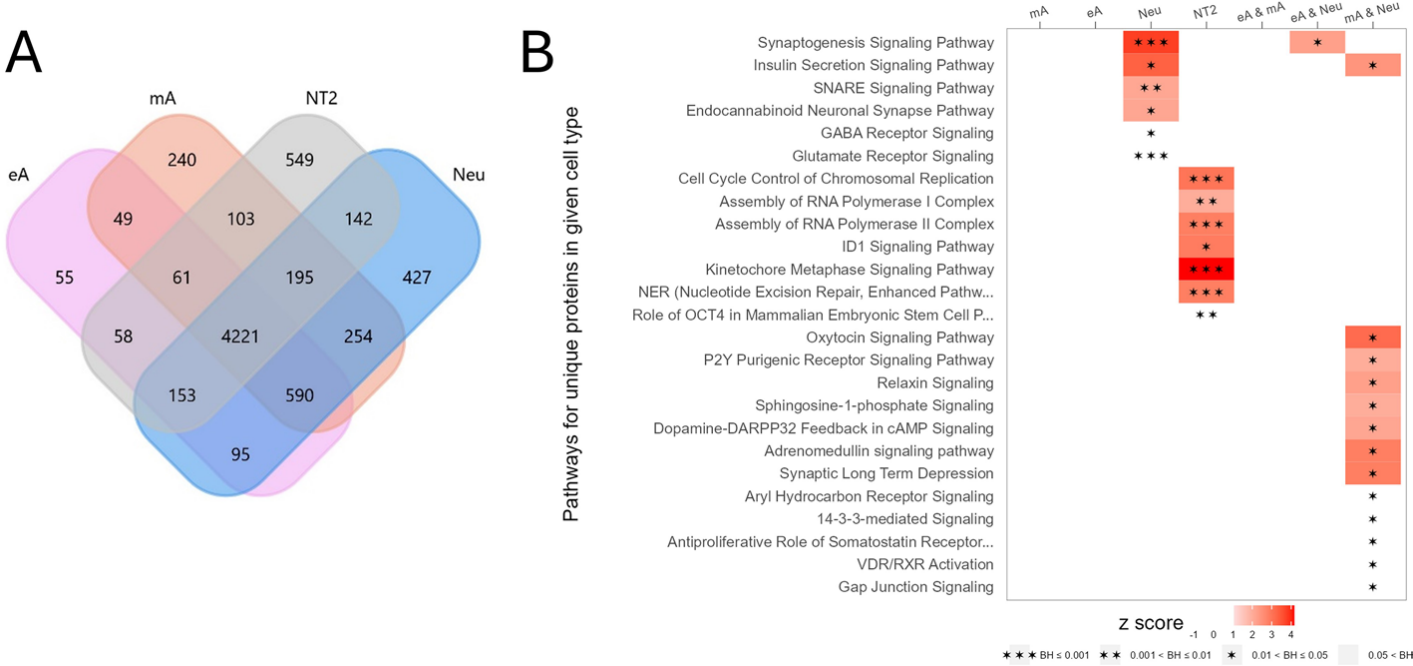
**A** Venn plot for reliably identified proteins, i.e., proteins that were present in at least 50% of samples in a group and for which the sum of the sequenced peptides was at least 130% of the number of samples in group. **B** Statistically significant canonical pathways for unique proteins in a given cell type, as obtained in IPA (*p*-value with Benjamini–Hochberg correction < 0.05)

Further analysis provided insights into a set of proteins unique to specific cell types (Fig. 3A). Ingenuity Pathway Analysis (IPA) database, used to identify such proteins, revealed upregulation of synaptogenesis and the insulin secretion signaling pathway and the presence of GABA and glutamate receptor signaling in Neu cells. In mA and Neu cells, the P2Y purinergic and aryl hydrocarbon receptor signaling pathways were enhanced (Fig. 3B). The analysis of molecular functions of proteins unique to Neu cells indicates characteristic neuronal morphology, synaptic functions, and intercellular communication. Furthermore, eA cells seemed to sustain transcriptional activity, whereas a notable feature of mA cells was the formation of cilia (Supplementary Fig. S10).

### Quantitative comparative analysis of proteomes across the different cell types

**Fig. 4:**
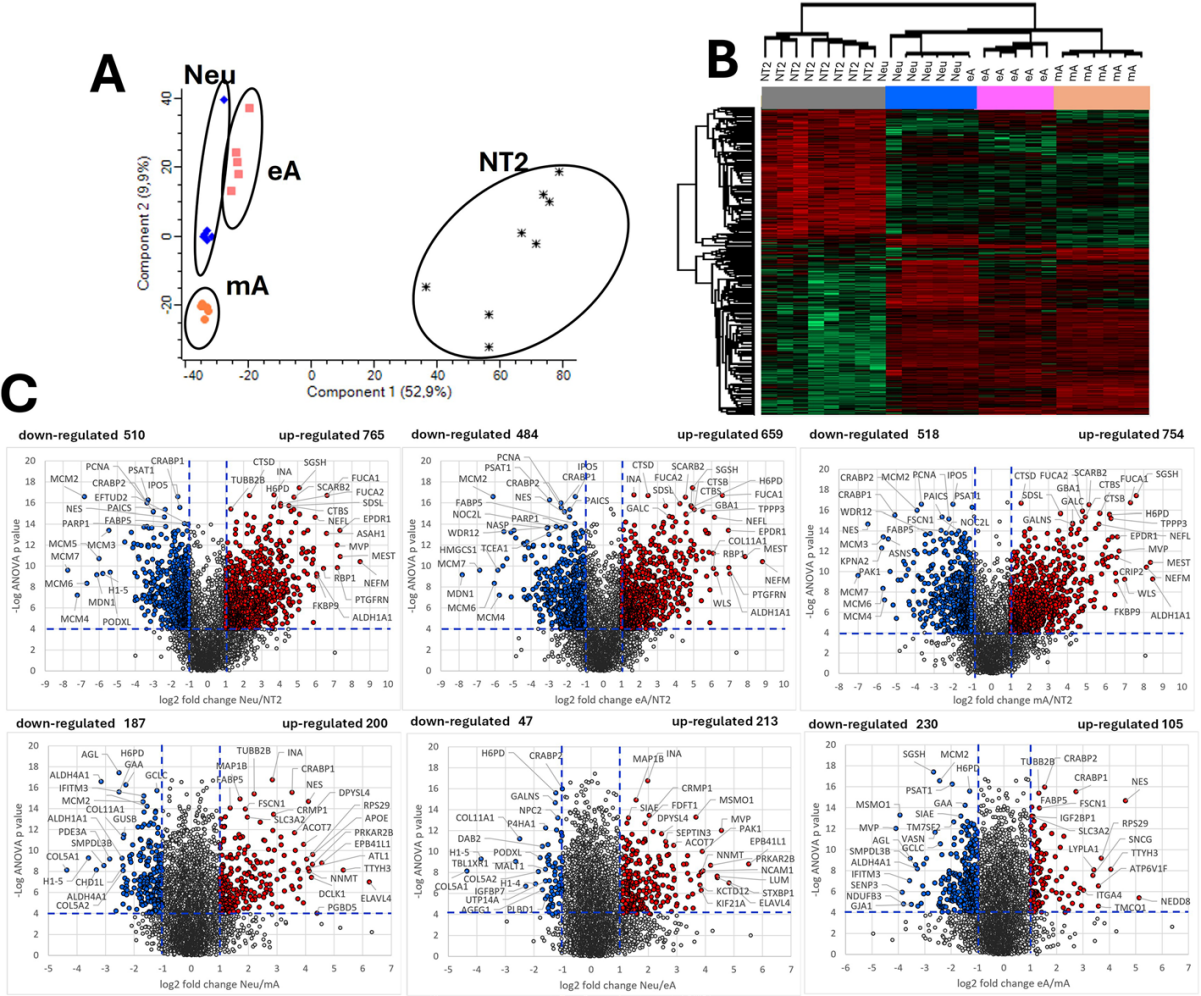
**A** Principal component analysis (PCA) of proteomic data [log_2_ label-free quantification (LFQ)] for the studied cell types. The PCA plot represents 3827 proteins (meeting the condition of having LFQ values present in 70% of all samples) with biological replicates, indicating clear proteomic profile differences between Neu (neuron-enriched), eA (early astrocytes), mA (mature astrocytes), and NT2 (undifferentiated) cells. **B** Hierarchical clustering and a heatmap. The 2145 proteins whose amount significantly differed between the four cell types [ANOVA permutation based false discovery rate (FDR) < 0.0001] are represented in the heatmap. **C** Volcano plots of the differentially expressed proteins (DEPs). The red and blue dots represent significantly upregulated and downregulated DEPs, respectively. The horizontal dotted line represents −log_10_ ANOVA *p*-value ≥ 4; vertical dashed lines indicate fold change |log_2_ FC|≥ 1

A comparative analysis of the proteins common to the four cell cultures studied revealed significant proteome alterations within the differentiated cells compared with the original NT2 cells (Fig. 4).

**Fig. 5:**
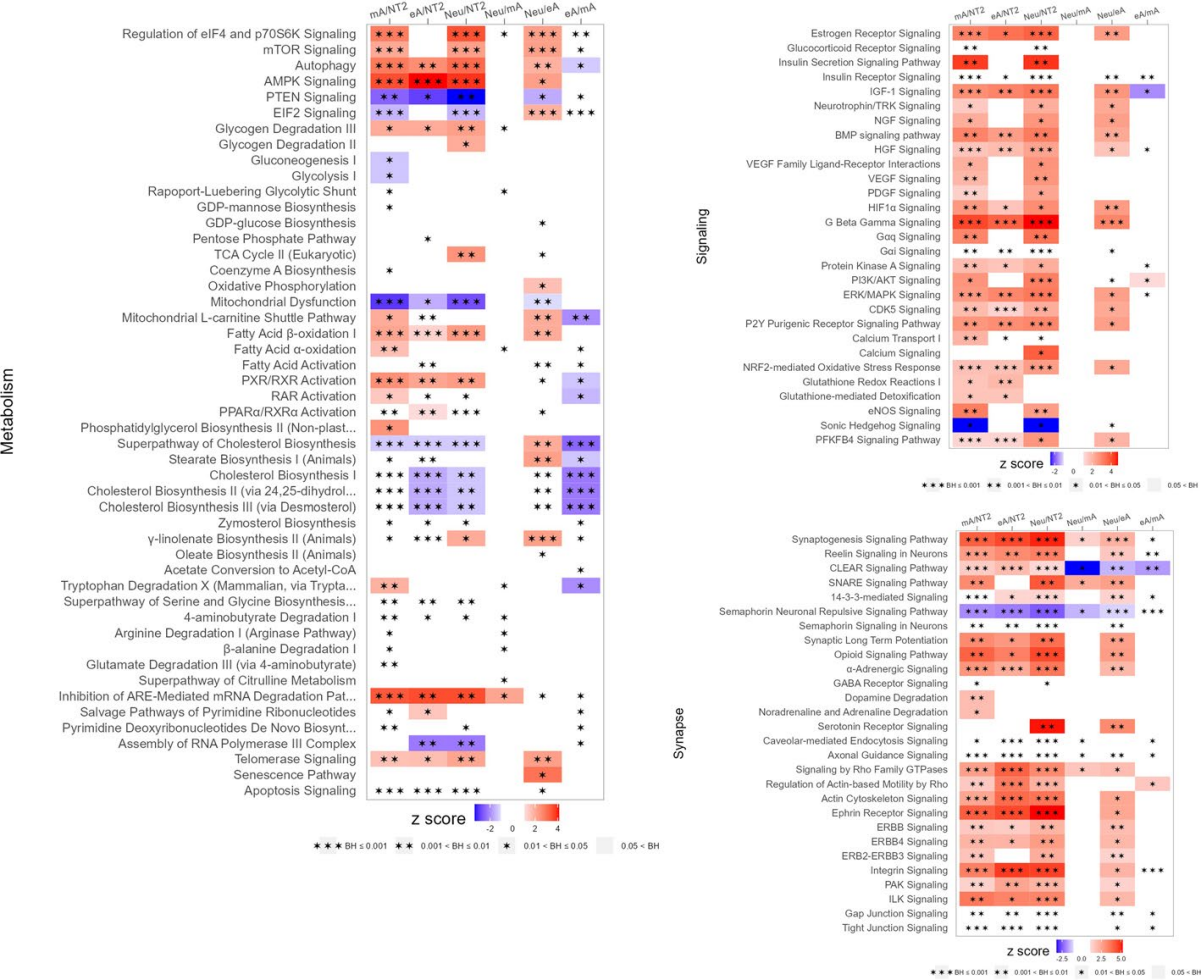
The most altered biochemical pathways clearly distinguishable using IPA analysis (*p*-value with Benjamini–Hochberg correction < 0.05) for DEPs meeting the ANOVA test (permutation-based FDR < 0.0001 and post hoc Tukey’s test in Perseus) and with a fold change (FC) > 2

The changes in biochemical pathways indicate a substantial metabolic rearrangement during the differentiation process in Neu and mA cells (and a less pronounced effect in eA cells), with upregulation of the mTOR pathway and enhancement of mitochondrial metabolism (Fig. 5). Additionally, in Neu and mA cells (but not in eA cells), we noticed enhanced insulin secretion signaling, as well as regulation of insulin and glucocorticoid receptor signaling, both of which play crucial roles in the differentiation of neurons and astrocytes (Fig. 5). Insulin signaling influences neuronal differentiation by regulating gene expression and controlling energy metabolism. Glucocorticoid receptor signaling affects the differentiation of neurons and astrocytes by regulating gene expression related to their function and survival, as well as synaptic plasticity and cognitive functions, which are essential for the proper nervous system development and function. Similarly, we observed a stronger upregulation of neurotrophic and growth factor signaling in Neu and mA cells than in eA cells. Upregulation of neurotrophin/TRK, NGF, VEGF, PDGF, and eNOS pathways is expected in neurogenesis and confirms the proper progression of neuronal development in the Neu culture. These pathways are intricately involved in neuronal survival, differentiation, and neuroprotection. The same signaling pathways are also involved in the process of astrocyte differentiation, contributing to their maturation, proliferation, and functional specialization (Fig. 5). Especially VEGF is important in astrocyte differentiation, influencing their morphology, proliferation, and function. All differentiated cells exhibited enhanced synapse-related signaling, but only Neu cells showed statistically significant upregulation of serotonin receptor signaling (Fig. 5).

**Fig. 6:**
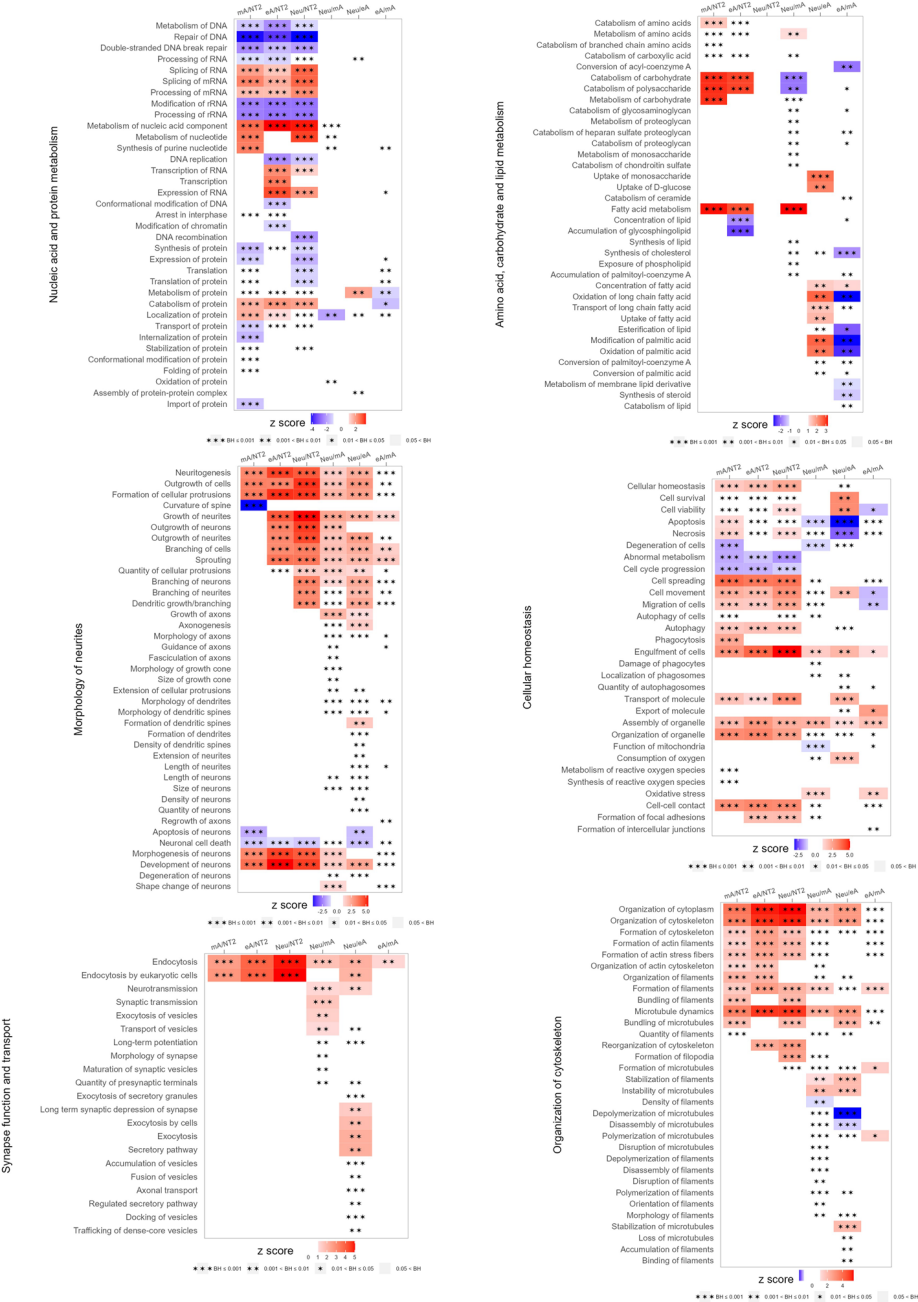
Major statistically significant molecular functions clearly distinguishable using IPA analysis (*p*-value with Benjamini–Hochberg correction < 0.05) for DEPs meeting the ANOVA test (permutation-based FDR < 0.0001 and post hoc Tukey’s test in Perseus) and with a fold change (FC) > 2

Alterations in molecular functions indicated a decline in nucleic acid and protein metabolism in the differentiated cells. However, nucleotide metabolism was enhanced in Neu and mA cells (Fig. 6), which is a rather expected finding, as nucleotides play a crucial role in neuron–glia communication [73]. The morphology of neurites in Neu cells was more intricate than in eA or mA cells, correlating with robust microtubule stabilization. Furthermore, synaptic functions and transport were most prominent in Neu cells (Fig. 6). In mA cells, there was significant involvement in phagocytosis, metabolism of reactive oxygen species, and cell-to-cell contacts (Fig. 6).

**Fig. 7:**
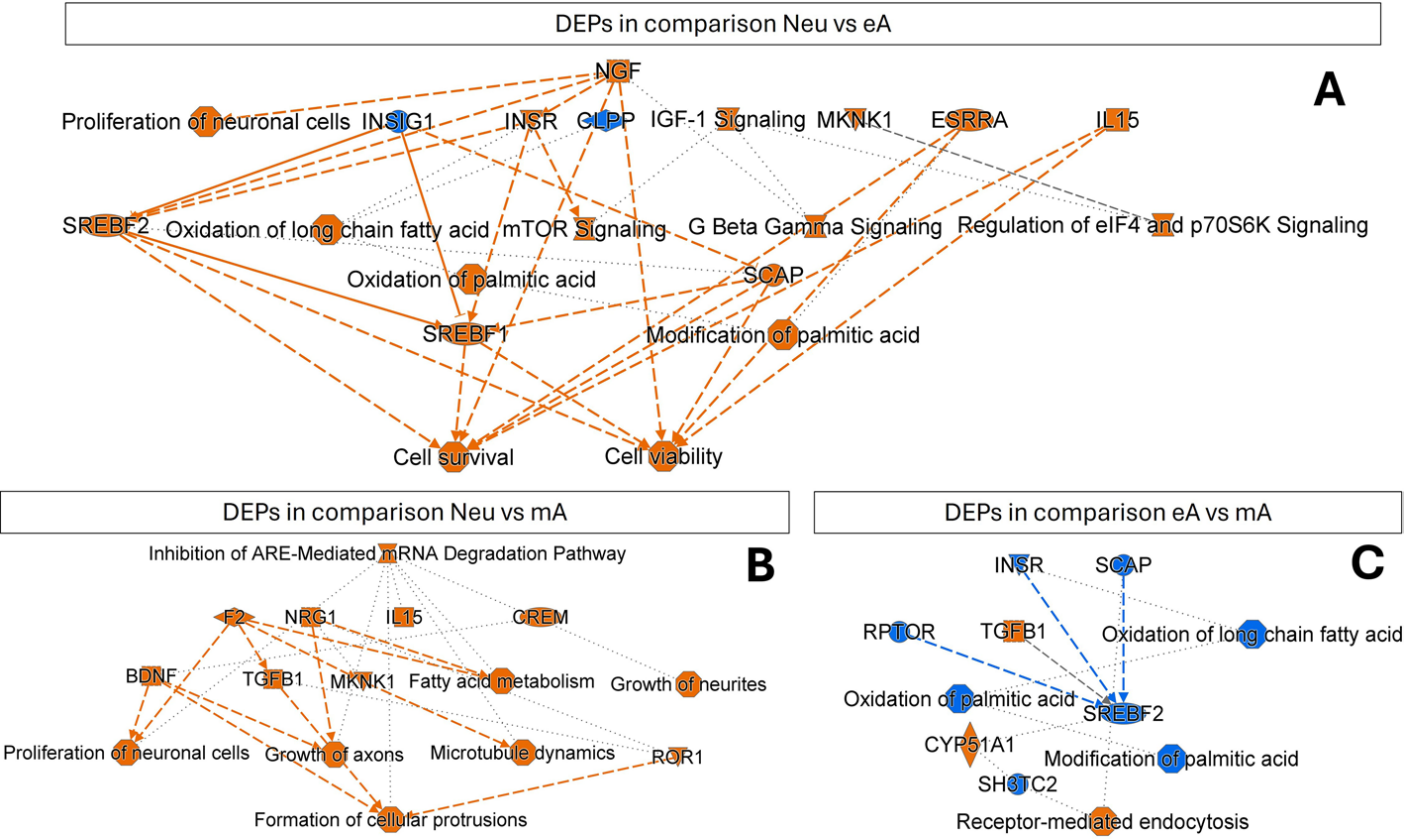
A machine learning-based, IPA-generated graphical summary of regulatory signaling for DEPs in the differentiated cells studied. The pictures illustrate biochemical pathways, cellular functions, and upstream regulators fulfilling *p* < 0.05, *z*-score ≥ 2. Orange and blue symbols indicate predicted activation and inhibition, respectively. The relationships between nodes are as follows: orange and blue lines indicate “leads to activation” and “leads to inhibition,” respectively. A solid line denotes direct interaction, a dashed line indicates indirect interaction, and a dotted line represents an inferred relationship

A comprehensive overview of proteins differentially expressed in Neu and eA cells (Fig. 7A) indicated the activation of NGF as a major upstream regulator, which had the following effects: (i) downregulation of insulin-induced gene 1 (INSIG1) resulting in the upregulation of transcription factors controlling cholesterol synthesis (SREBF1, SREBF2, and SCAP), (ii) upregulation of insulin receptor (INSR) resulting in the activation of mTOR signaling, and (iii) downregulation of caseinolytic protease (CTPP) involved in the degradation of misfolded proteins and the regulation of mitochondrial protein synthesis rate. Additionally, the activation of insulin-like growth factor-1 (IGF-1) and estrogen-related receptor alpha (ESRRA) subsequently enhanced cell survival and viability.

Functional variances in cells within the Neu culture, as opposed to mA cells (Fig. 7B), were influenced by BDNF, F2 (prothrombin), and its downstream regulators such as TGF-β1, MKNK1, as well as by NRG1 (neuregulin). The activation of these regulatory proteins is likely a secondary effect of the increased stability of Arc (activity-regulated cytoskeleton-associated protein) and of the activation of ROR1 protein. Arc is causally implicated in increased long-term potentiation (LTP) and decreased long-term depression (LTD), and homeostatic synaptic scaling [74]. ROR1 is highly expressed in cultured postmitotic mature astrocytes and promotes fatty acid metabolism [75].

In eA, compared with mA astrocytes (Fig. 7C), the observed downregulation of fatty acid metabolism resulted from the downregulation of mTOR signaling and transcription factors related to cholesterol metabolism. Additionally, receptor-mediated endocytosis, which was upregulated in eA cells, is presumably controlled by SH3TC2, an important regulator of the endocytic recycling pathway [76].

### Electrophysiology of neurons

**Fig. 8:**
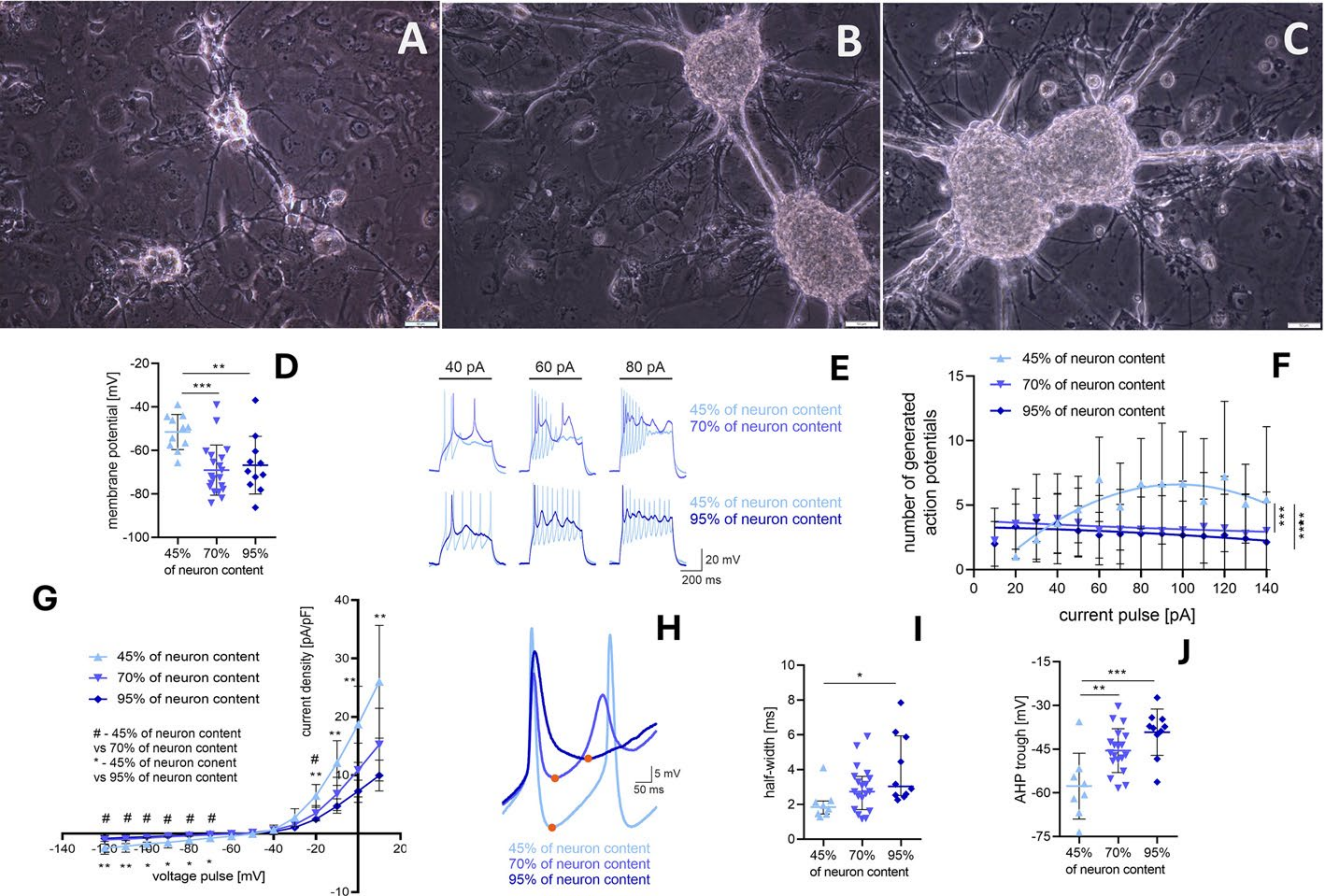
Upper panel: Representative images of neurons cultured in the presence of varying amounts of astrocytes. **A**–**C** represent 45%, 70%, and 95% neuron content in a culture, respectively. Scale bars always indicate 50 µm. Lower panel: Electrophysiological properties of neurons: resting membrane potential (**D**), excitability (**E, F**), current density at different voltages (**G**), shape of action potential induced by a +80 nA (**H**): half-width pulse (**I**) and value at the lowest point of afterhyperpolarization (marked with orange dot in **H**) (**J**). In **G** hash signs indicate results of post hoc tests between 45% and 70% neuron content, and asterisks indicate results of comparisons between 45% and 95% neuron content

Whole-cell voltage and current patch-clamp recordings were used to characterize neuron electrophysiological properties in the cultures with different neuron to astrocytes ratios (Fig. 8A–C). Differences in the proportions of neurons to astrocytes reflect the natural variability of the cell culture. Results revealed that the neuron’s resting membrane potential in the culture that contained 45% neurons was the least hyperpolarized, and it significantly differed from that of cultures containing either 70% or 95% neurons (Fig. 8D).

There were no differences between the tested groups in other passive membrane properties, i.e., capacitance, resistance, or time constant (Supplementary Fig. S11). At the same time, neurons of the cultures containing 45% neurons exhibited the most physiological excitability. They responded to increasing current pulses with a greater number of action potentials, and differed from the two remaining groups, in which the number of action potentials remained relatively constant regardless of stimulation intensity (Fig. 8E, F). Voltage step stimulation of the examined neurons revealed strong outward rectification of the recorded current in all tested groups. At the same time, amplitudes of the outward whole-cell current recorded for neurons of the culture containing 45% neurons, were significantly higher in the voltage range of −20 to +20 mV, relative to the group containing 70% of neurons. Inward current amplitudes were also higher in the culture containing 45% neurons, and they differed significantly in the voltage range of −120 to −70 mV from currents recorded for the culture with 95% neurons (Fig. 8G). To evaluate possible differences in the characteristics of action potential generated by the tested neurons, the shape of a single action potential evoked by a depolarizing current pulse was compared (Fig. 8H). Results revealed that that neurons of the cultures containing 45% neurons had the narrowest AP, and their AP half-width differed significantly from that of neurons in the 95% neuron culture (Fig. 8I). Moreover, in neurons of the culture with a 45% neuron content, the AHP trough voltage reached the most hyperpolarized values, and differed significantly from AHP trough of neurons of the two other cultures (Fig. 8J). The remaining AP properties did not differ between the tested groups (Supplementary Fig. S11), although a consistent trend was preserved.

Taken together, these observations clearly demonstrate that a significant ratio of astrocytes to neurons (about 1:1) is essential for achieving physiological maturity in neurons. This is in line with previous reports that showed that culturing NT2-derived neurons on primary astrocytes enabled the neurons to form functional glutamatergic excitatory and GABAergic inhibitory synapses [77].

The Neu culture had a similar composition (60% neurons and 40% astrocytes) to the culture whose neurons in patch clamp studies showed electrophysiological features characteristic of healthy mature neuronal cells. The inspection of protein content in the Neu cultures, in terms of cell excitability and electrophysiological properties of neurons, revealed the presence of all the principal protein groups involved in these functions, i.e., voltage-gated calcium channels (CACNA1E, CACNA1C, CACNA2D1, CACNA2D2, CACNB1, CACNB3, CBARP, and related PKD2); voltage-gated potassium channels (KCNA6, KCND2, KCNB2, KCNIP1, and related DPP6); other potassium channels; voltage-gated sodium channel (SCN3B); voltage-gated chloride channels (CLCC1, CLCN3, CLCN5, CLCN6, and CLCN7); ligand-gated ion channels; glutamate receptors (GRIA2, GRIA4, GRID1, and related PICK1), GABA receptors (GABRA3, GABRB3) and glycine receptor (GLRA2); sodium/potassium ATPases pumps (ATP1A1, ATP1A3, ATP1B1, and ATP1B3), calcium ATPase (ATP2B2); sodium-calcium exchanger (SLC8A1); sodium/hydrogen exchangers (SLC9A1, SLC9A6, and SLC9A7); ion transporters/exchangers (SLC4A7, SLC4A8, SLC12A2, SLC12A4, SLC12A7, SLC12A8, and SLC12A9); neurotransmitter transporters including that of glutamate (SLC1A2 and SLC1A3); GABA (SLC6A1); other monoamine transporters (SLC6A15 and SLC6A17); proteins involved in synaptic vesicle trafficking and neurotransmitter release (i.e., SCL17A6/VGluT2, SLC18A3/VAChT, SLC32A1/ VGAT, SNAP25, STX1A, VTI1B, and STX7), calcium signaling proteins, and proteins related to receptor modulation and signal. Many of these proteins were unique to the Neu culture, and the vast majority of the remaining ones were found at their highest levels in Neu cells (Supplementary Table S6).

The protein profile of Neu cells confirmed that NT2-derived neurons, in the presence of an appropriate number of astrocytes, were able to reach a proper maturation state and perform their functions effectively.

## Discussion

In this study, we determined the identity of neurons in co-cultures with astrocytes (i.e., 60% neurons and 40% astrocytes) through in-depth proteome profiling. Additionally, we present, for the first time, a comprehensive protein profile of NT2-derived astrocytes at two time points during their maturation process and in a neuron-enriched culture. Our analysis included the examination of astrocytic markers, from which we inferred maturity and reactivity status of astrocytes in these three culturing regimes. Furthermore, we explored the functionality of neurons growing in co-cultures with varying ratios of astrocytes. With maturity, neurons in neuronal cultures often exhibit a more hyperpolarized membrane potential; however, in our current study, we observed that the neuron’s resting membrane potential in the 45% Neu culture was the least hyperpolarized of all the groups examined. At the same time, neurons from 45% Neu culture had most physiological excitability and action potential properties, characteristics that are critical indicators of neuronal functionality and maturation in vitro. This allows us to assume that the recorded value of the membrane potential was a characteristic property of the neurons that matured in this culture.

### Beneficial effects of astrocytes on neuronal physiological features: insights from electrophysiology

Electrophysiological studies using whole-cell voltage and current techniques showed that neurons flourish when cultured in a balanced environment with astrocytes. In a culture containing 45% neurons to 55% astrocytes, the neurons showed excitability and action potential shape characteristic of mature neurons. Optimal ratio of neurons to astrocytes not only facilitated better electrophysiological responses but was also associated with a robust protein profile, characterized by high levels of essential proteins that are key to neuron action potential, synaptic function, and neuronal signaling, thus confirming the functional competence of these neurons. These findings underscore the fundamental role of astrocytes in enhancing neuronal maturation and function. A balanced content of astrocytes is essential for reaching a mature state and effectively performing physiological functions by neurons, further highlighting the importance of astrocytes in the overall health and functionality of neuronal networks.

Importantly, several proteins that were detected exclusively in the Neu cultures play a critical role in shaping the functional properties of neurons. Particularly noteworthy is the expression of the following ion channels proteins: potassium voltage-gated channel subfamily D (KCND2) involved in neuronal cell membrane repolarization and frequency-dependent spike broadening [78], calcium voltage-gated channel subunit alpha1 E (CACNA1E) involved in firing pattern modulation in neurons [79], voltage-gated chloride channels mediating Cl^−^ currents [80] and contributing to electrical excitability and ion homeostasis [81] that, along with many other proteins identified in the Neu cultures in this study, play a crucial role in regulating membrane potential and action potential firing.

Notably, proteins implicated in neurotransmission, ion channel function, and synaptic plasticity, such as VGLUT2, GLUR2 or GABRA3, and GABRB3, were also detected in Neu cultures. VGLUT2 is a vesicular glutamate transporter responsible for loading l-glutamate into synaptic vesicles in excitatory neurons. The protein also contributes to glutamatergic neurotransmission [82]. Importantly, increased VGLUT2 expression may enhance synaptic transmission and influence neuronal excitability [83]. Similarly, the observed high expression of GLUR2 that encodes AMPA receptor subunit GluR2 as well as high expression of GABRA3 and GABRB3 involved in GABAergic signaling, in Neu cultures, indicate possible functional maturity of these neurons, as these proteins control synaptic plasticity and neuronal excitability [84]. Moreover, the presence of GLUR2 as well as GABRA3 and GABRB3 proteins indicates the Neu culture neuron capacity to form both excitatory and inhibitory synapses. This finding is further strengthened by the expression of cell adhesion molecules (such as CNTNAP2, CNTNAP2, and NRCAM) mediating cell–cell interactions and synapse formation. In addition, the presence of pH regulators (such as SLC4A7/NBCn1, SLC4A8/NDCBE1, and SLC9A6) is another indication of a proper development and maturity of neurons in the Neu culture, as proper pH regulation is essential for maintaining neuronal homeostasis and excitability [85].

The proteins observed in Neu culture validated the neuronal capacity for generating and transmitting electrical impulses.

### Synergistic interplay between neurons and astrocytes

A particularly intriguing observation is that astrocytes cultured with neurons (Neu culture), despite being 3 weeks younger than mA cells, exhibited a protein expression profile consistent with that of mature astrocytes. Therefore, not only is the maturation time crucial, but also the intercellular contacts both between astrocytes themselves and between astrocytes and neurons enable astrocytes to gain a characteristic phenotype. Astrocyte–astrocyte contact is a powerful factor promoting astrocyte maturation [86], therefore the density of an astrocyte culture is one important maturation stimulus. However, a mutual interaction between astrocytes and neurons appears to be crucial for effective functioning of both cell types. For instance, it was demonstrated that immature neurons co-cultured in the presence of NFIA-induced astrocytes show evidence of accelerated maturation through increased expression and appearance of SYN1. NFIA-induced astrocytes also promote neuronal survival when subjected to glutamate excitotoxicity [39]. Conversely, PAX6 was shown to contribute to neuron-dependent GLT-1 induction in astrocytes [56]. Moreover, in astrocytes co-cultured with neurons, the induction of many astrocytic genes, including GLT-1, occurs upon receiving neuron-derived, Notch-dependent maturation signals [87], as in our Neu culture. Astrocytes can emit both positive and negative signals (i.e., SPARCLE1/SPARC) for synapse formation, thus modulating neuronal action. Additionally, estrogen produced by astrocytes acts as an important synapse-stimulating factor [88]. Cholesterol, a precursor to steroid hormones, is also produced by astrocytes, which supply both cholesterol and sterols to neurons [89].

In this study, the estrogen receptor signaling pathway was the most strongly activated pathway in the differentiated cells (and particularly in the Neu culture) in comparison with NT2 cells. Moreover, estrogen receptor signaling was significantly more pronounced in the Neu culture than in the eA culture.

### Astrocytes

In contrast to the results of staining for GFAP, all eA and mA astrocytes were GLUL- and VIM-positive. GFAP expression is known to be induced during astrocyte maturation [90] or activation [91]. In terms of the progression of GFAP expression during astrocytic maturation, Western blotting revealed a significant increase in the GFAP level within the 6-week maturation period following the completion of RA treatment. A detectable level of GFAP protein was reached by the second week of maturation (Fig. 1C).

However, in the immunochemistry experiment on eA cells (third week of maturation), only a very small population (1–2%) of astrocytes exhibited GFAP-positive staining (Fig. 1B e, g). In the 6-week-old mA culture, the subset of GFAP-positive cells was larger, but it did not exceed 30–40% (Fig. 1B i, j).

Notably, previous reports showed less than 5% of intense GFAP-positive cells in NT2-derived cells immediately after RA treatment [43]. In a 4-week-old maturating astrocyte culture, intense GFAP staining was previously observed in a subset of cells (up to 45%), and it concerned smaller fibrous astrocytes, and not the large protoplasmic cells [8, 92–95]. Some authors reported a complete absence of GFAP and GLUL/GS expression from NT2-derived adherent cells corresponding to protoplasmic astrocytes (with diameters exceeding 100 µm), even after stimulation with LPS. This inconsistency could be attributed to differences in culturing conditions [96].

### The GFAP issue

Although GFAP is the most widely used astrocytic marker, not all astrocytes do express GFAP [97] [98]. Moreover, GFAP antibody labels the thick main processes of only a subset of astrocytes, predominantly in the white matter. In most astrocytes of the cortical gray matter, GFAP is not detectable [38, 99]. Fibrous astrocytes display lower basal metabolism [100] and a probably increased susceptibility to adverse conditions [101]; they also show lower viability, and have limited proliferative capacity, which could explain why, in a culture that matured for more than 4 weeks in this study, mainly protoplasmic astrocytes persisted. In this study, the KI67 level in the differentiated cultures was lower than in the NT2 cells (but there were no significant differences in its amount between eA and mA), which confirmed low proliferative potential of the differentiated cells. Another issue is that GFAP level can vary between fewer than 100 copies and 50,000 copies of mRNA per an individual astrocyte of primary astrocyte cultures, as shown by single-cell gene expression profiling [102]. We also noticed substantial variability in the GFAP level caused by even small differences in culture density or replating frequency.

Notch signaling in neural stem/progenitor cells (NPCs) has been implicated in maintaining undifferentiated, activated NSCs (NOTCH1) and quiescent NSCs (NOTCH2 and NOTCH3), and also in inhibiting neuronal differentiation and promoting differentiation into an astrocyte lineage [103]. Notch signaling is context- and tissue-dependent. Activated NOTCH1 and NOTCH3 promote astroglial differentiation from multipotent progenitors in CNS [104], although NOTCH1 is now recognized as a central regulator of astrogliosis in neuro-inflammation [105]. NOTCH2 (but not NOTCH1) is strongly expressed in postnatal mouse glial cells that express high levels of VIM and low levels of GFAP [106] [107]. In our study, NOTCH1 was exclusively detected in NT2 cells, confirming their undifferentiated character. NOTCH2 level was higher in eA than in mA or Neu, and it was lowest in NT2 cells, while NOTCH3 was present in all cell types, with the highest levels observed in Neu cells (Supplementary Table S2).

Importantly, Notch signaling mediates bidirectional astrocyte–neuron communication [108]. Astrocytes control the neurogenic niche and inhibit neuronal differentiation of neural NPCs via Notch signaling. This process depends on cytoplasmic intermediate filaments (IFs), specifically GFAP and VIM. Therefore, the expression of these proteins is tightly temporally regulated. The absence of both GFAP and VIM (but not of GFAP only or VIM only) from astrocytes leads to decreased Notch signaling from astrocytes to NPCs and increased neuronal differentiation of NPCs [109, 110]. On the other hand, neurons induce astrocytic differentiation of NPCs by triggering Notch signaling, leading to the demethylation of astrocyte-specific gene promoters, such as NFIA, and subsequently promoting GFAP expression [111]. This could explain our observation of strong GFAP-positive staining in the vicinity of neurons in the Neu culture.

Generally, the proteome of eA cells was more similar to that of Neu cells than to the mA proteome (Fig. 4A, B). Indeed, immature astrocytes were shown to be similar to neuronal cells [112]. It was even demonstrated that astrocytes possess a latent neurogenic potential, and that blocking Notch signaling triggers astrocytes to enter a neurogenic program [113].

One of the most important regulators of GFAP expression is the STAT3 signaling pathway. STAT3 levels were low in undifferentiated NT2 cells, they were higher in Neu, and they significantly increased in eA and mA cells. STAT3 binds to the GFAP promoter and promotes DNA demethylation, resulting in GFAP expression, which is important in the early stage of astrocytogenesis [114]. Conversely, SIN3A, which was found at its highest level in NT2 cells, binds to the GFAP promoter and represses GFAP transcription. However, upon differentiation, STAT3 binding removes SIN3A from the promoter and activates GFAP expression [115]. On the other hand, FGF2 induces the expression of GFAP via ciliary neurotrophic factor (CNTF, an IL-6 cytokine family member) [116], possibly by recruiting STAT3 [117]. We found the highest level of FGF2 in mA, followed by a relatively high level in eA and a lower level in Neu. The lowest level of FGF2 was detected in NT2 cells. Recently, leukemia inhibitory factor (LIF, an IL-6 cytokine family member) has been recognized as the most efficient factor in generating GFAP-positive cells compared with other glial differentiation factors [39]. In our study, LIF was detected only in the Neu culture. In the healthy CNS, LIF is almost exclusively expressed in neurons, specifically the cholinergic and GABAergic ones; it induces neurogenesis and neurite outgrowth [118]. Thus, LIF regulates the generation of new neurons and their supporting glial cells and is another example of a neuron–astrocyte interplay mediator.

Considering the composition and the abundance of marker proteins reflective of astrocytes (as depicted in the overview in Fig. 2), along with the biochemical pathways and molecular functions affected by the sets of uniquely and differentially expressed proteins in eA and mA cultures, it became evident that mA cells display a more extensive profile of marker proteins compared with eA cells.

### Relevance and limitations of the study

The closer characterization of astrocytes derived from the Ntera-2 cell line and their co-culture with neurons presented herein allows for broader utilization of this line in a range of neuroscience research. This includes studies on molecular mechanisms characteristic of astrocytes, through mechanisms of intercellular communication exampled in works by Sandhu’s team [119, 120], to advanced research on the mechanisms of reprogramming astrocytes to neurons in the context of neurological and neurodegenerative diseases. For instance, astrocytes and neurons derived from NT2 cell line were used to study the role of PTBP1 and REST proteins in the mechanism of reprogramming astrocytes to neurons [121, 122]. The NT2-derived astrocyte model holds significant potential for studying the mechanisms of transdifferentiation from reactive astrocytes (induced by ischemia) into mature neurons. Specifically, by targeting PAX6 [123], Notch signalling and NFIA/B/X [124] or DLX2 [125] but also other transcription factors or microRNA [126], this approach could provide crucial insights necessary for developing new strategies to treat ischemic injuries. Astrocytes are of particular interest owing to their proliferative response to injury or neurodegeneration. Reactive astrocytes represent a pool of reprogrammable cells within the adult mouse brain. Additionally, reactive astrocytes play a crucial role in various beneficial processes during the recovery phase following nervous system injuries [127].

While most studies have been conducted using mouse models, it is important to note that human astrocytes differ significantly from those of mice. The strong predominance of rodent-derived markers (mouse: 55; rat: 35 of total 111 in Supplementary Table S2) in the current literature highlights a critical gap in human-specific cell reference data. This underlines the importance of employing human-relevant models such as NT2 to expand the available marker repertoire and improve our understanding of astrocyte identity in the human context. Therefore, NT2-derived astrocytes serve as a valuable model for advancing neuroscience research.

The proteomic characterization of NT2-derived astrocytes cultured under traditional serum-supplemented conditions, as presented in this work, may aid in designing experiments involving reprogramming experiments [128]. We have provided information on culture-specific transcription factors and regulatory proteins that could be utilized in testing new hypotheses. Despite being cultured in serum-supplemented medium as described in earlier established protocols, the obtained astrocytes did not show a significant increase in reactivity protein markers, notably GFAP, as was extensively discussed. Nonetheless, the culturing conditions influenced their functionality, rendering them particularly effective for studying specific aspects of reactivity. Particularly interesting in the context of reprogramming are early astrocytes derived from the Ntera-2 cell line (eA), which upon manipulation can give rise to mature neurons.

NT2 cells should be considered as a model with certain limitations, as is the case with all models. Alternative, the hiPSC model also presents its own challenges, including variability between donor individuals, issues with genetic stability, and experimental inconsistencies, all of which can impact differentiation capacity, cellular heterogeneity, morphology, as well as transcript and protein levels. NT2 cells were chosen for their capacity to differentiate into both neurons and astrocytes. This simultaneous differentiation better reflects physiological conditions compared with human iPSCs, which require distinct sets of growth factors for each cell type. In contrast, NT2 cells undergo parallel differentiation of both cell types under retinoic acid treatment, offering a model that more closely resembles the in vivo environment. Moreover, iPSC-directed differentiation typically yields cell populations that resemble fetal or neonatal stages, and obtaining mature, electrically active neurons requires coculture with astrocytes [129]. On the other hand, determining the maturity of both neurons and astrocytes on the basis of a single marker can be misleading, as is the case with GFAP. Therefore, it is advisable to consider a set of markers, as demonstrated in this study.

While this study successfully identifies several candidate markers for neuronal subpopulations via in-depth proteomics, a notable limitation is the absence of orthogonal validation through methods such as immunocytochemistry. Confirmation of these promising proteomic candidates will be essential and is a designated objective for our future follow-up studies.

## Conclusions

This study explored the status of astrocytes at two stages of maturation and in two distinct culture settings, i.e., pure cultures and co-cultures with neurons. Comprehensive analyses were conducted on the protein profiles of these astrocytes, and the protein profile of neurons in neuron-enriched cultures was also portrayed. Additionally, the functionality of neurons was assessed across cultures with varying neuron-to-astrocyte ratios. The study also investigated key astrocytic regulatory proteins, including STAT3, FGF2, and LIF, a range of transcription factors, and the role of intermediate filaments such as GFAP, alongside other markers reported in the literature. The findings highlight a substantial reciprocal interplay between astrocytes and neurons, with significant communication facilitated by secretory proteins, such as THBS2, TNC, SPARCL1, and SPARC, as well as by signaling pathways, including TGF-β, Notch, insulin, and estrogen. Collectively, the findings highlight the importance of astrocyte–neuron co-cultures as a model that more adequately represents the inherent properties of neural tissue.

## Supplementary Information

Additional file 1.

Additional file 2.

Additional file 3.

Additional file 4.

Additional file 5.

Additional file 6.

Additional file 7.

Additional file 8.

Additional file 9.

## Data Availability

All data supporting reported results are available at the following address: https://doi.org/10.57903/UJ/VEDN1K

## Abbreviations

ABC: Ammonium bicarbonate
AC: Cytosine arabinoside
ACN: Acetonitrile
ACSF: Artificial cerebrospinal fluid
ANOVA: Analysis of variance
AP: Action potential
BPs: Biological processes
BRUBs: Britton–Robinson universal buffers
BSA: Bovine serum albumin
CAPS: 3-(Cyclohexylamino)-1-propanesulfonic acid
CNS: Central nervous system
DEP: Differentially expressed proteins
DIA: Data-independent acquisition
DMEM: Dulbecco’s modified Eagle’s medium
DTT: Dithiothreitol
Early A, eA: Early astrocytes
Enriched N, Neu: 60% Of neurons and 40% of astrocytes
ER: Endoplasmic reticulum
FASP: Filter-assisted sample preparation
FBS: Fetal bovine serum
FDR: False discovery rate
FdU: Fluorodeoxyuridine
GO: Gene Ontology
HG: High glucose
hiPSCs: Human induced pluripotent stem cells
IFs: Intermediate filaments
IPA: Ingenuity pathway analysis
LC MS/MS: Liquid chromatography–tandem mass spectrometry
LFQ: Label-free quantification
LPS: Bacterial lipopolysaccharides
LTD: Long-term depression
LTP: Long-term potentiation
Mature A, mA: Mature astrocytes
MS: Mass spectrometry
NPCs: Neural stem/progenitor cells
NSCs: Neural stem cells
PBS: Phosphate-buffered saline
PIPs: Phosphoinositides
PVDF: Polyvinylidene fluoride
RA: All-trans retinoic acid
RPs: Reactome pathways
RT: Room temperature
RuBPs: Ruthenium (Tris(bathophenanthrolinedisulfonate))
SAX: Strong anion exchange
SDS: Sodium dodecyl sulfate
SDS-PAGE: Sodium dodecyl sulfate–polyacrylamide gel electrophoresis
TFA: Trifluoroacetic acid
U: Uridine
