# Supplementary material for: NT2-derived astrocyte–neuron co-culture reflects physiological relevance and offers research validity
Source: Cell Mol Biol Lett. 2025 Jul 25;30:90. doi: 10.1186/s11658-025-00765-z (PMC12291249; doi:10.1186/s11658-025-00765-z)
Supplement: Supplementary file 1 — Additional file 1. [file 11658_2025_765_MOESM1_ESM.pdf]

Ntera-2-derived Astrocyte-Neuron Co-Culture Reflects Physiological Relevance and Offers Research Validity

Sylwia Kedracka-Krok[1*], Ewelina Fic[1], Zuzanna Cepil[1], Piotr Rybczyński[1], Agata Szlaga[2], Radosław Cacała[1], Sławomir Lasota[3], Anna Blasiak[2], Marta Dziedzicka-Wasylewska[1]

[1]Jagiellonian University, Faculty of Biochemistry, Biophysics and Biotechnology, Department of Physical Biochemistry, Krakow, Poland (sylwia.kedracka-krok@uj.edu.pl; ewelina.fic@uj.edu.pl; zuzanna.cepil@uj.edu.pl; piotr.rybcznski@student.uj.edu.pl; radoslaw.cacala@student.uj.edu.pl; marta.dziedzicka-wasylewska@uj.edu.pl)

[2]Jagiellonian University, Institute of Zoology and Biomedical Research, Kraków, Poland (agata.szlaga@alumni.uj.edu.pl; anna.blasiak@uj.edu.pl)

[3]Jagiellonian University, Faculty of Biochemistry, Biophysics and Biotechnology, Department of Cell Biology, Kraków, Poland (slawomir.lasosta@uj.edu.pl)

*Correspondence:
Sylwia Kedracka-Krok
sylwia.kedracka-krok@uj.edu.pl

Supplementary material

Table S1 List of antibodies used in immunocytochemistry and Western Blot experiments

**Antibody**	**Specification**			
**Primary**	Type	Manufacturer	Catalogue number	**Dilution**
anti-NeuN	monoclonal rabbit	Cell Signaling Technology	24307S	1:50
anti-GFAP	monoclonal rabbit	Cell Signaling Technology	80788S	IF 1:200<br>WB 1:2000
anti-GFAP	monoclonal mouse	Cell Signaling Technology	3670S	1:300
anti-FABP7	monoclonal rabbit	Cell Signaling Technology	13347S	1:250
anti-vimentin	monoclonal rabbit	Cell Signaling Technology	5741S	1:100
anti-synapsin I	polyclonal rabbit	Invitrogen	A6442	1:500
anti-GS	monoclonal rabbit	Invitrogen	701989	1:500
anti-ezrin	monoclonal mouse	Invitrogen	35-7300	1:250

anti-MAP2	monoclonal mouse	Sigma-Aldrich	M4403	1:500

Antibody	Specification			
anti-rabbit	AF 488 goat IgG	Invitrogen	A-11008	IF 1:500
anti-mouse	AF 546 goat IgG	Invitrogen	A-11003	IF 1:500
anti-rabbit IgG (H+L)	Alexa Fluor Plus 647	Invitrogen	A32733	WB 1:1000
anti-rabbit	IgG (H+L) Cross-Adsorbed Secondary Antibody, HRP	Invitrogen	A16104	WB: 1:4000

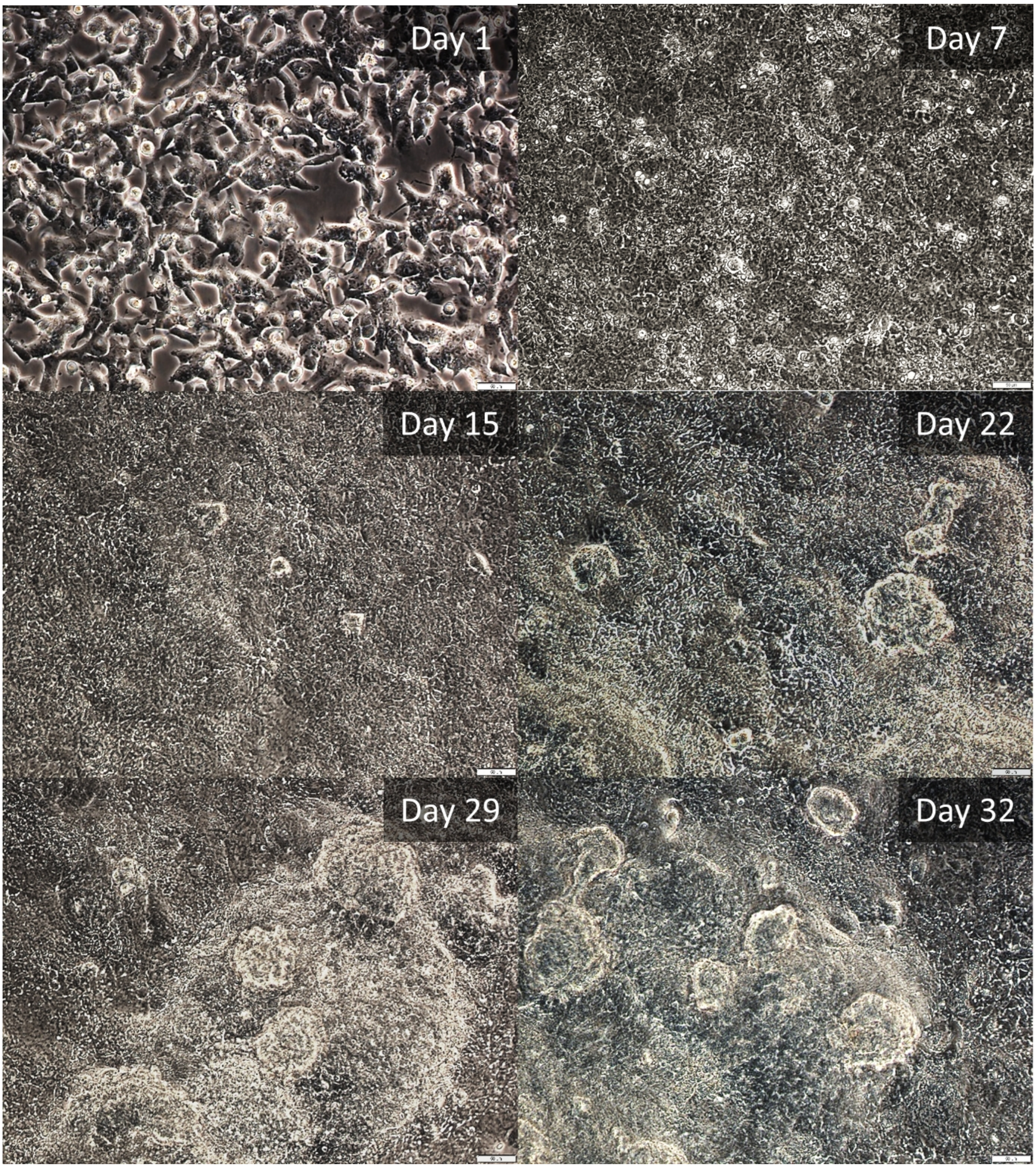

Fig. S1 **NT2 cell culture under 4 weeks differentiation procedure with all-trans retinoic acid**. Addition of retinoic acid and uridine three times a week, from day 1 and day 8, respectively, at final concentrations in the cell medium of 10 µM. The first

spheres were observed after three weeks (approx. day 21). The last photo was taken before cell passage (day 32). Microscope: Olympus IX81 with IX2-UCB-2 control module and QImaging MicroPublisher 3.3 RTV camera. Objective lens: 10x. Observation method: phase contrast. Scale bar: 50 µm

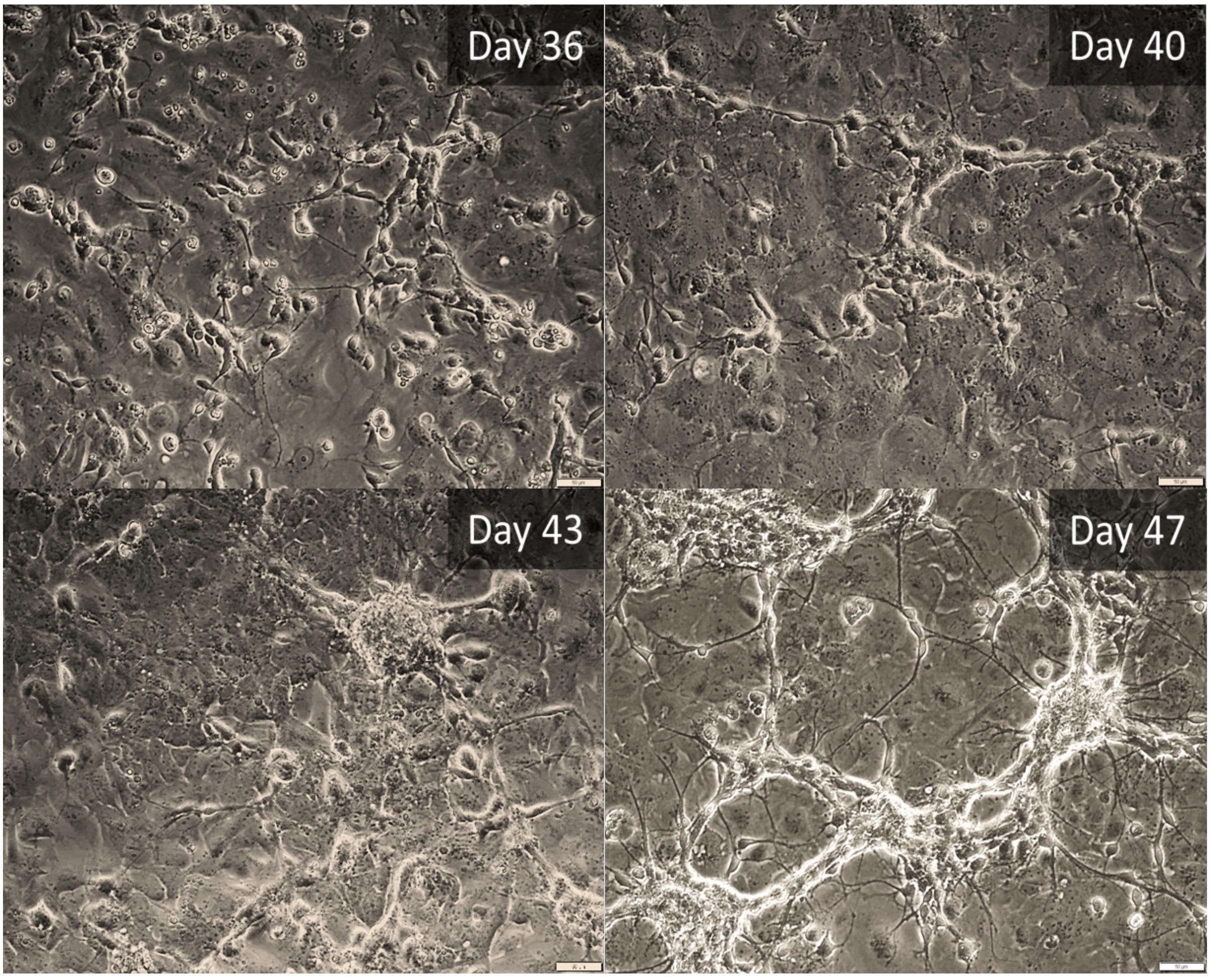

Fig. S2 **Maturation of separated less adherent cells – culture enriched in neurons (Neu).** Microscope: Olympus IX81 with IX2-UCB-2 control module and QImaging MicroPublisher 3.3 RTV camera. Objective lens: 10x. Observation method: phase contrast. Scale bar: 50 µm

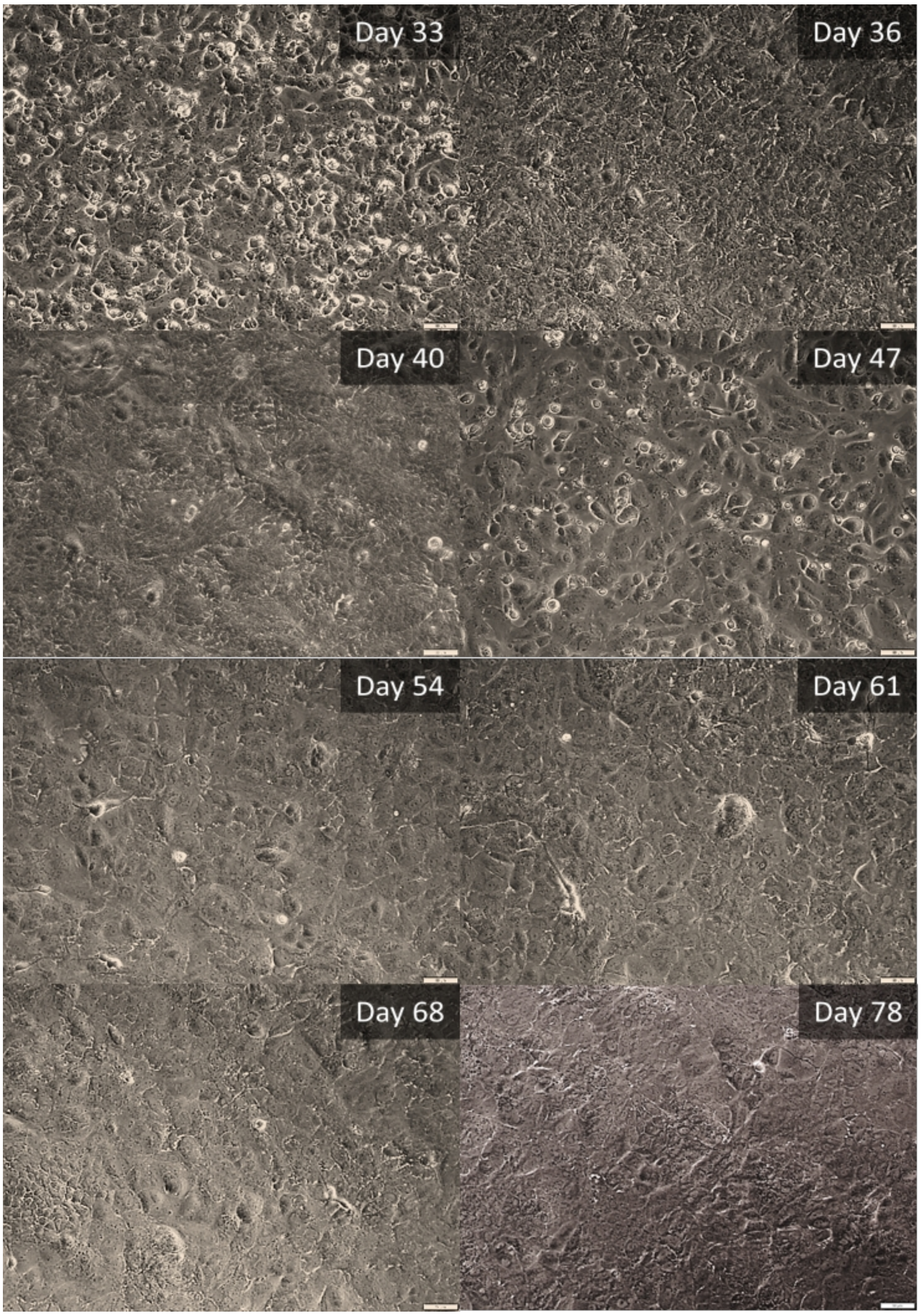

Fig. S3 **Maturation of separated more adherent cells – culture of astrocytes.** Microscope: Olympus IX81 with IX2-UCB-2 control module and QImaging MicroPublisher 3.3 RTV camera. Objective lens: 10x. Observation method: phase contrast. Scale bar: 50 µm

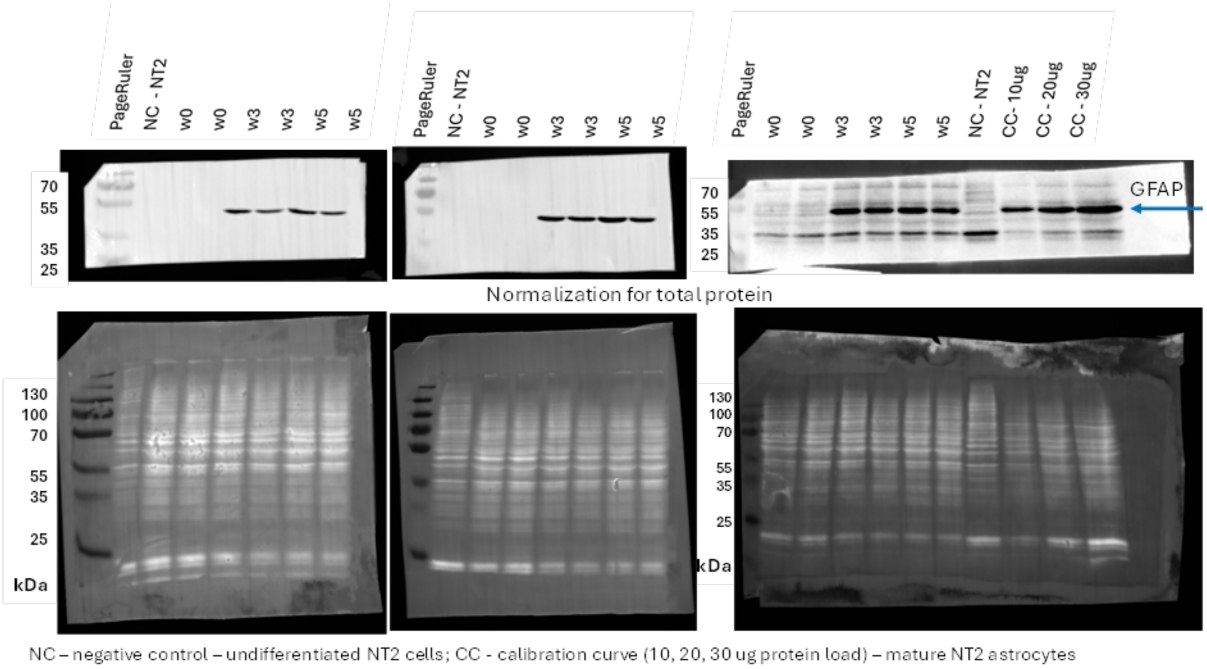

Fig. S4 **Time-dependent monitoring of GFAP protein expression through Western Blot analysis**: Upper panel: full-size Western Blot membrane detecting GFAP by luminescence. Lower panel: fluorescence staining of the entire membranes using Ruthenium dye. w0, w3 and w5 denote weeks of astrocyte maturation. w0 denotes the starting point of maturation after the completion of retinoic acid treatment. The analysis was conducted across four biological replicates. Normalization was done for total protein load after fluorescence statning with Ruthenium dye

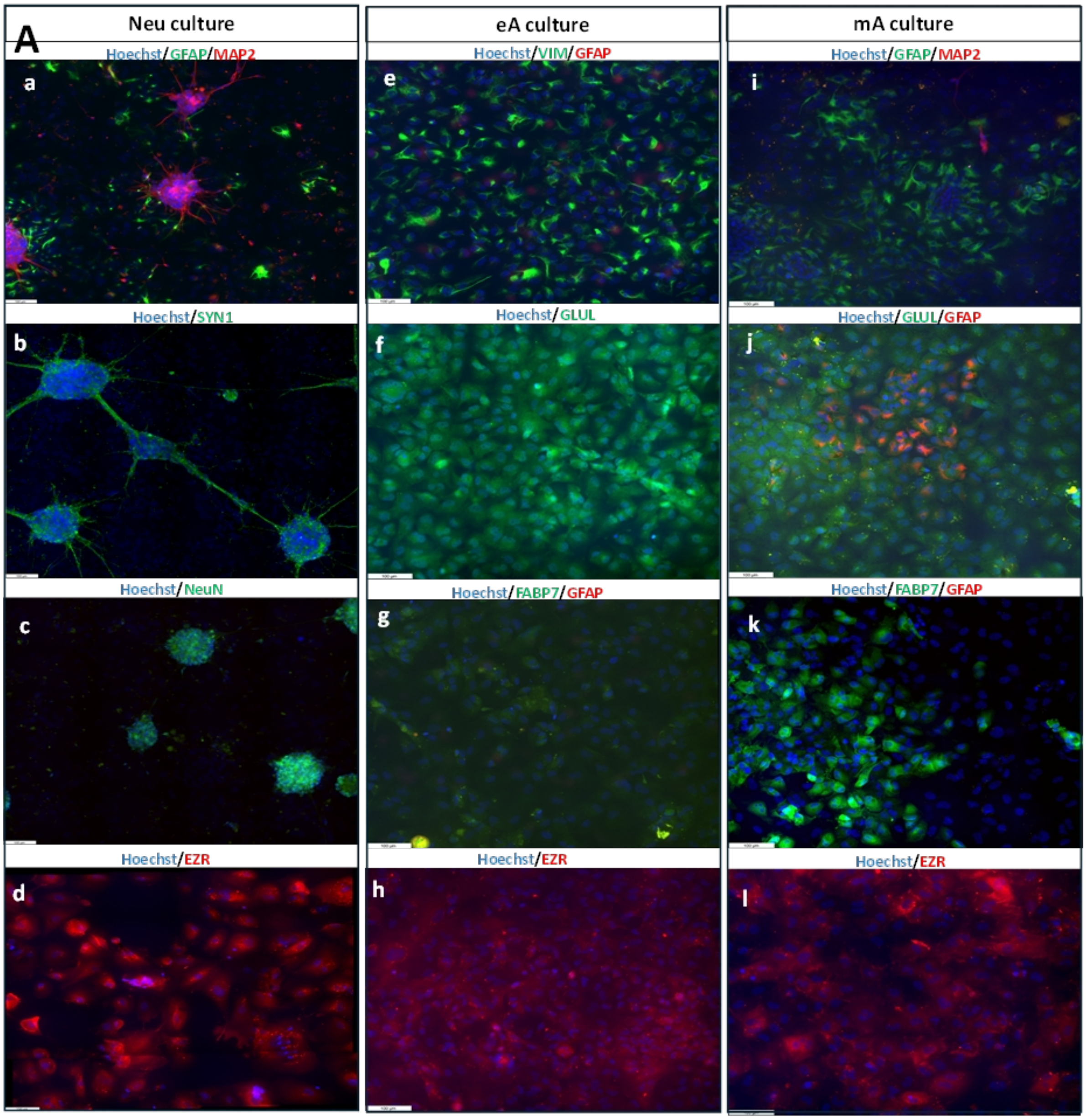
A
Neu culture
eA culture
mA culture
Hoechst/GFAP/MAP2
Hoechst/VIM/GFAP
Hoechst/GFAP/MAP2
a
e
i
Hoechst/SYN1
Hoechst/GLUL
Hoechst/GLUL/GFAP
b
f
j
Hoechst/NeuN
Hoechst/FABP7/GFAP
Hoechst/FABP7/GFAP
c
g
k
Hoechst/EZR
Hoechst/EZR
Hoechst/EZR
d
h
l

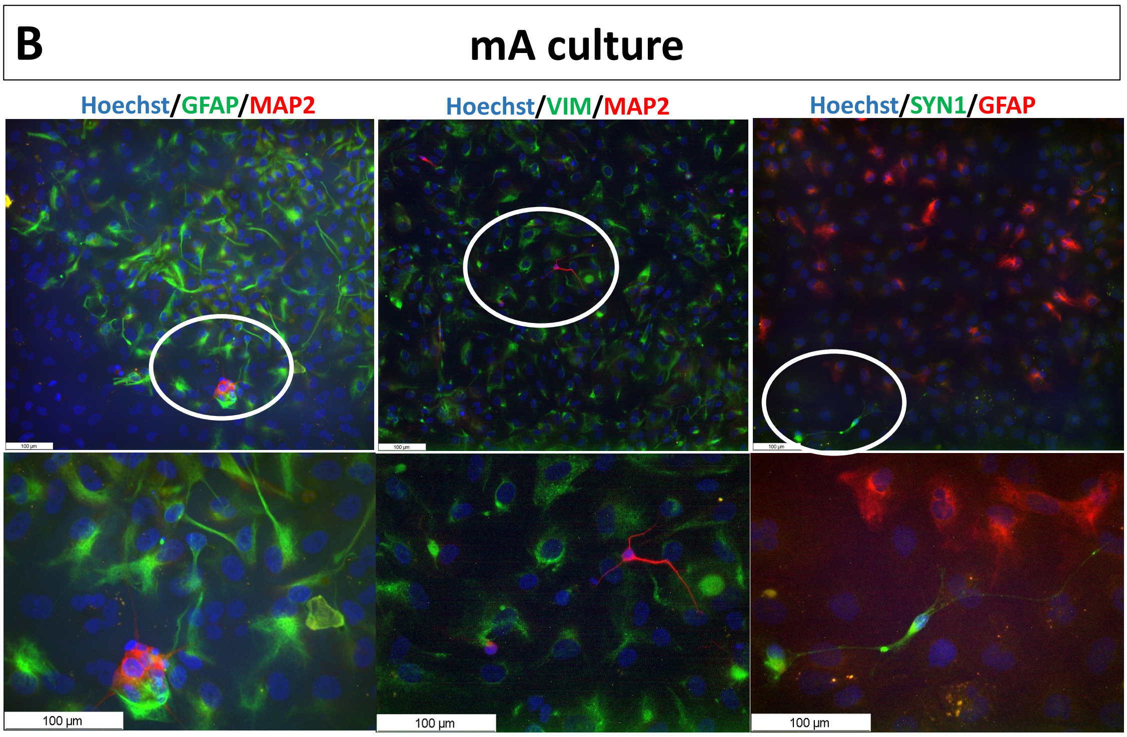
B
mA culture
Hoechst/GFAP/MAP2
Hoechst/VIM/MAP2
Hoechst/SYN1/GFAP
100 µm
100 µm
100 µm

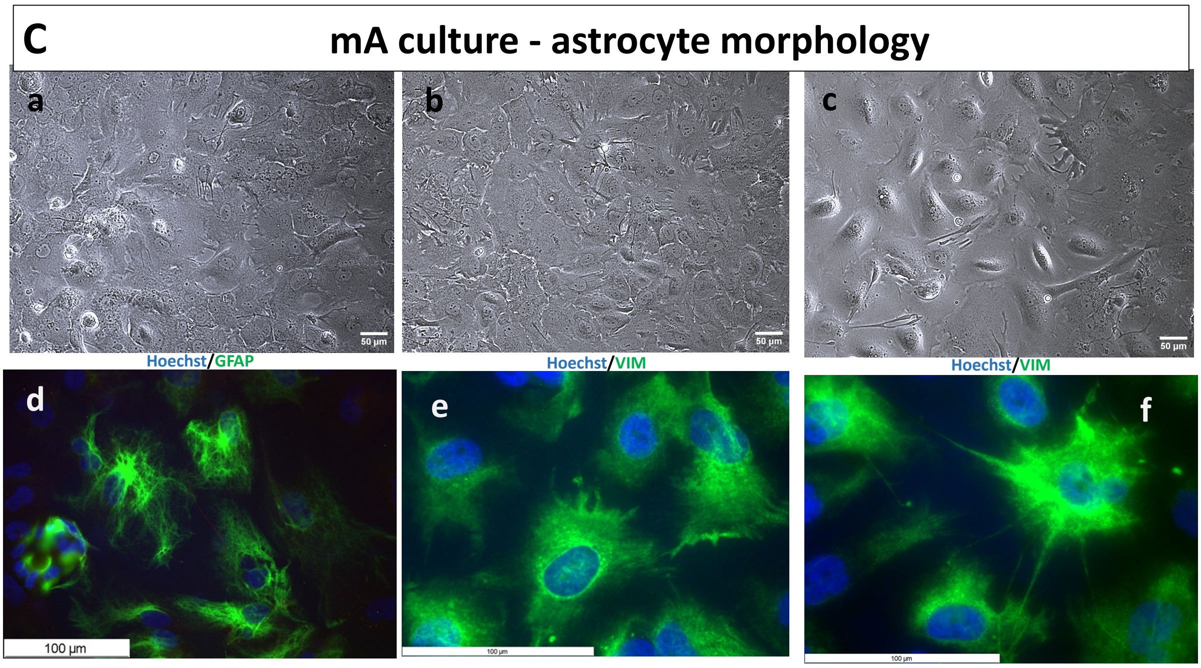
C
mA culture - astrocyte morphology
a
b
c
50 µm
50 µm
50 µm
Hoechst/GFAP
Hoechst/VIM
Hoechst/VIM
d
e
f
100 µm

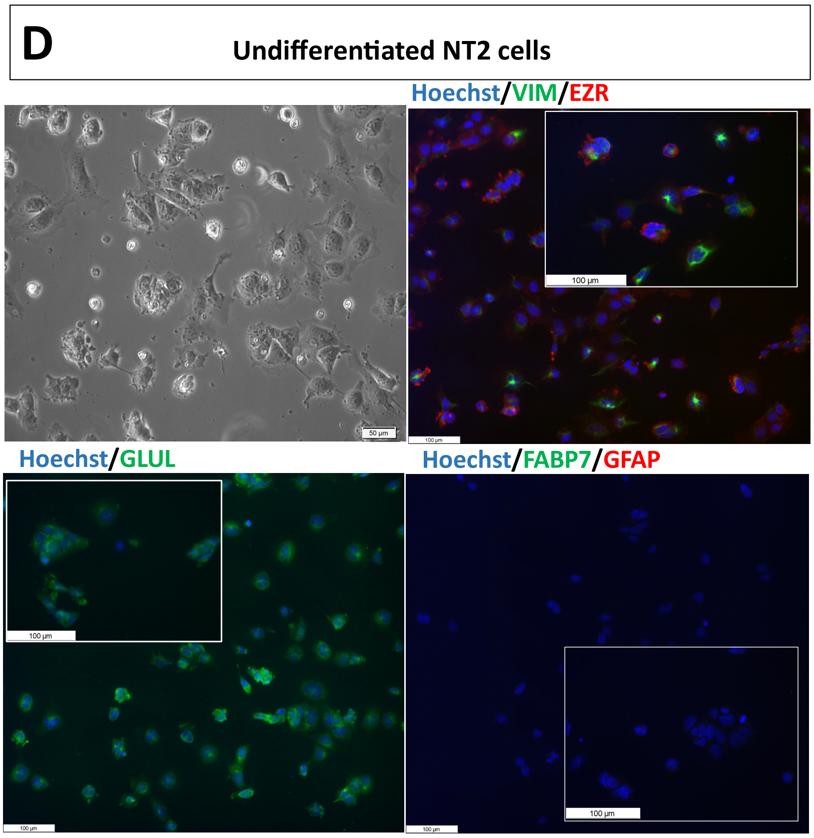

Fig. S5 (**A**) **Immunocytochemical staining for neuronal and astrocytic markers**, (**B**) **Neuronal markers in mA cultures**. The lower panel is a magnified view of the area marked with a circle in the images from the upper panel. (**C**) **Morphology of astrocytes in mA culture**. Phase contrast images; objective magnification: 10×, scale bars indicate 50 µm (a-c). Immunocytochemical staining for astrocytic (GFAP, VIM) markers. Nuclei are stained with Hoechst. Scale bars indicate 100 µm (d-f); objective magnification: 40× in d; 63× in e, f (**D**) **Immunocytochemical staining of undifferentiated NT2 cells** for neuronal and astrocytic markers, Scale bars: 50 µm for phase contrast images and 100 µm for fluorescence images, respectively. Each tile scan consists of 20 images

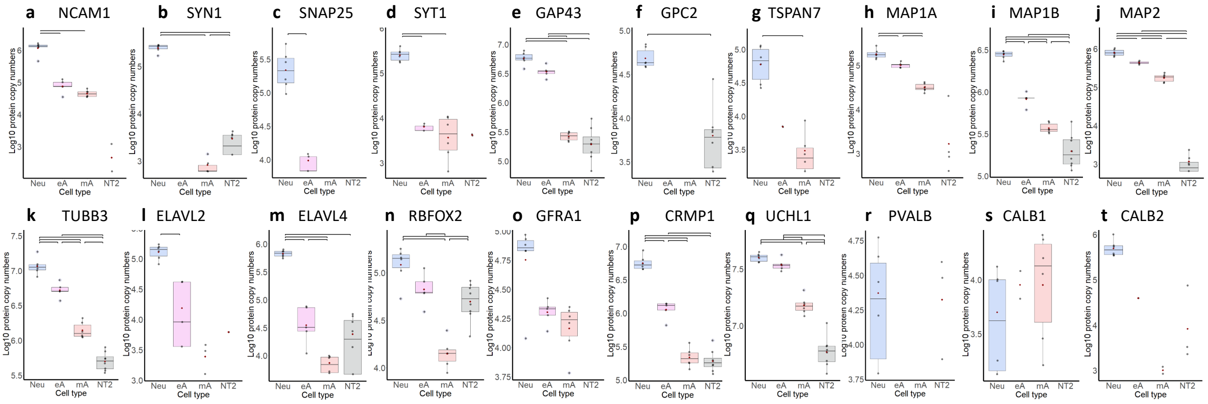

Fig. S6. **Neuronal protein copy number profiles in the examined cell types**, i.e: Neu (neuron-enriched), eA (early astrocytes), mA (mature astrocytes), and NT2 (undifferentiated) cells. Black lines indicate statistically significant differences, as determined by ANOVA with post-hoc Tukey test, or, in the cases of non-parametric conditions (checked by Shapiro-Wilk test), by Kruskal-Wallis and Mann-Whitney/Wilcoxon tests with Benjamini-Hochberg correction. In the event of missing values: if the proportion of missing values within a group was no more than 40%, then those missing values were replaced with the minimum value for that group. Box is signed when protein was identified in at least 60% samples in group. Red dot means average value in group

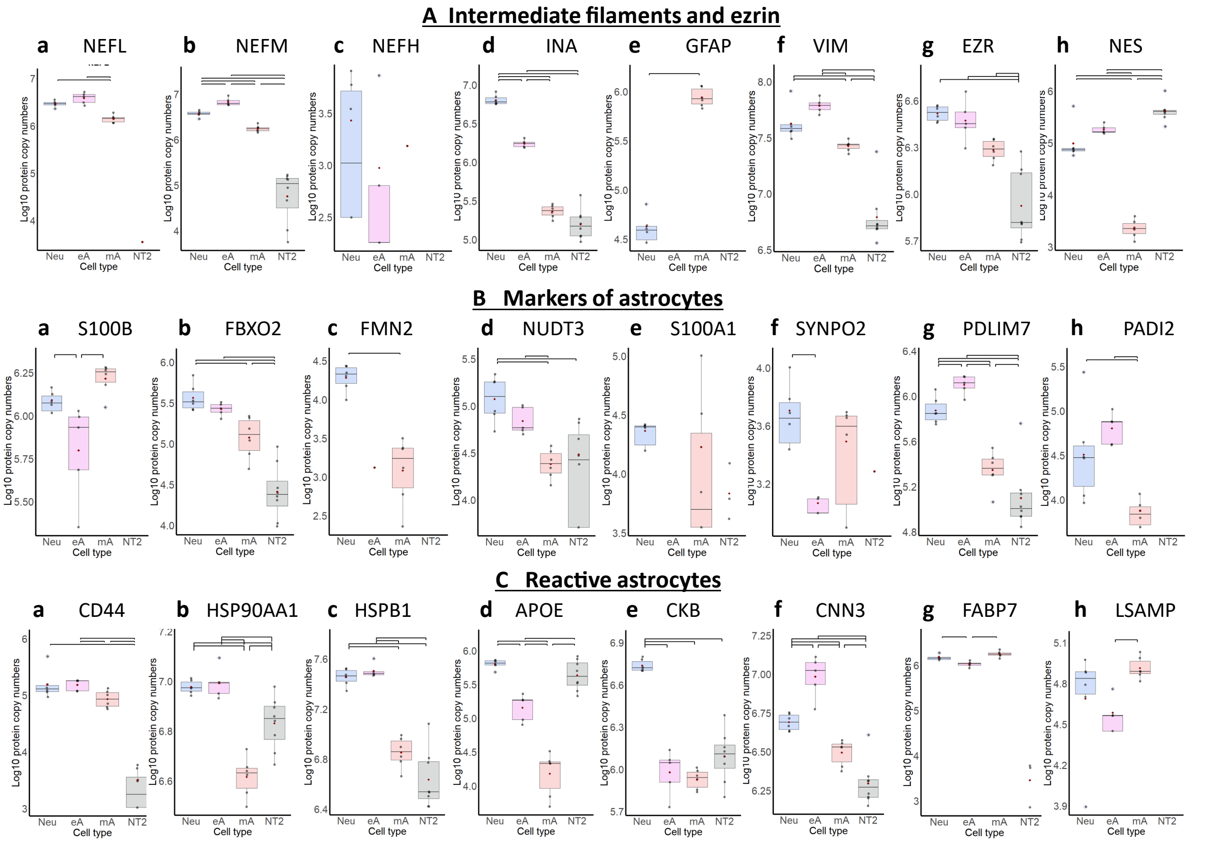

Fig. S7. **Protein copy number profiles in the examined cell types - IFs (A), astrocytic (B), reactive astrocytic (C) in** Neu (neuron-enriched), eA (early astrocytes), mA (mature astrocytes), and NT2 (undifferentiated cells)**.** Black lines indicate statistically significant differences as determined by ANOVA with post-hoc Tukey test, or, in the cases of non-parametric conditions (checked by Shapiro-Wilk test), by Kruskal-Wallis and Mann-Whitney/Wilcoxon tests with Benjamini-Hochberg correction. In the event of missing values: if the proportion of missing values within a group was no more than 40%, then those missing values were replaced with the minimum value for that group. Box is signed when protein was identified in at least 60% samples in group. Red dot means average value in group

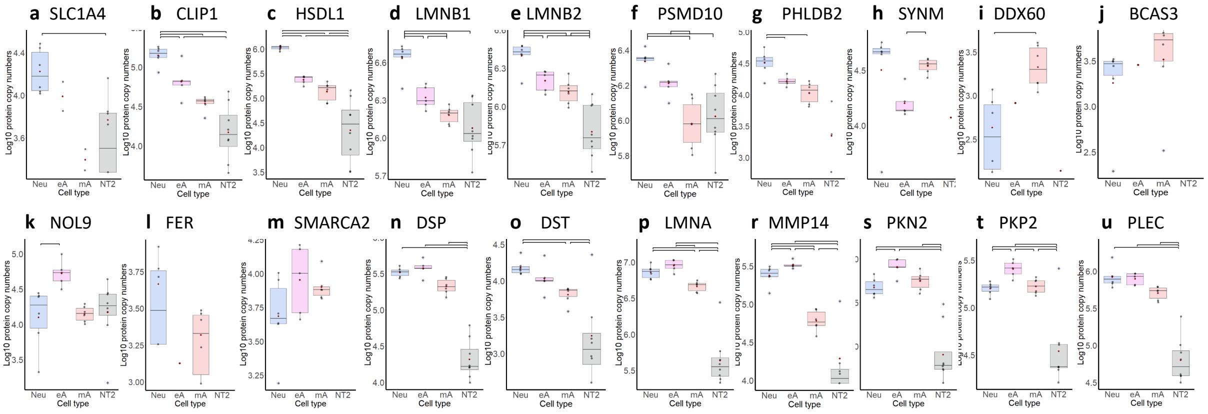

Fig. S8 **Levels of cellular intermediate filaments in examined cell types.** Black lines indicate statistically significant differences as determined by ANOVA with post-hoc Tukey test, or, in cases of non-parametric conditions (checked by Shapiro-Wilk test), by Kruskal-Wallis and Mann-Whitney/Wilcoxon tests with Benjamini-Hochberg correction. In the event of missing values: if the proportion of missing values within a group is no more than 40%, then those missing values are replaced with the minimum value for that group. Box is signed when protein was identified in at least 60% samples in group. Red dot means average value in group

Some proteins belonging to IFs were predominantly present in the Neu culture, including NRP1, NDEL1, SESTD1, IFFO1 (Table S2). NRP1 (neuropilin-1) is a protein present in presynaptic terminals, where it serves as a ligand-binding receptor for class 3 semaphorins which promote dendritic growth and branching in adult hippocampus [1]. SESTD1 (SEC14 and spectrin domain-1) is also involved in dendrite, spine, and synapse formation [2].

Other IFs highly expressed in Neu culture included CLDN11 (Fig. S9 o), SCL1A4/ASCT1, CLIP1, HSDL1, LMNB1, LMNB2, PSMD10/gankyrin, PHLDB2, and SYNM (Fig S8 a-h). Some of these proteins classified as IFs have important regulatory functions. SLC1A4 is a major D-serine uptake system in astrocytes and it can also export L-serine via heteroexchange, supplying neurons with the substrate for D-serine synthesis. Deletion of SLC1A4 is associated with neurodevelopmental alterations linked to changes in the relative contributions of D-serine vs. glycine in mediating NMDA receptor activity [3]. PSMD10 is a proteasome assembly chaperone and an oncoprotein, but it also facilitates human neural precursor cells (NPCs) differentiation to neurons via the β-catenin/Ngn1 pathway [4]. PHLDB2 in neurons regulates synaptic plasticity, the turnover of the glutamate receptor, and dendritic spine morphology [5]. SYNM, whose highest level was observed in Neu cells (Fig. S8 h), is thought to be present in immature or reactive astrocytes, but an isoform of SYNM can also be expressed by human neurons [6]. In mA, there were high amounts of DDX60 and BCAS3 (Fig S8 i, j). VIM and EZR were detected in relatively high quantities in eA and Neu cells (Fig. S7A f, g). In eA cells, there was a high amount of S100A1 (Fig. S7B a) and NOL9 (Fig. S8 k). EZR does not belong to IFs, but it physically interacts with GFAP and GLAST to control the abundance of GLAST in the plasma membrane [7]. Additionally, the absence of EZR leads to the upregulation of GFAP and a transition to reactive astrocyte phenotype [8]. In NT2 and eA cells, a high level of NES (nestin, Fig. S7A h), an IF classified as a neural progenitor cell (NPC) marker, was observed. NES is considered relevant to the reactive state of cells, but it has also been found to be present in a subset of unchanged astrocytes. Furthermore, NES negatively regulates adult neurogenesis through notch signaling [9].

A relatively high level of NOL9 was found in eA cells, but the role of this protein in astrocytes is unknown (Fig. S8 k). There were other IFs whose levels increased significantly in the differentiated cells compared to the NT2 cells, namely FER, SMARCA2, DSP, DST, LMNA, MMP14, PKN2, PKP2, and PLEC (Fig. S8 l-u), which are all engaged in adhesion and cell-cell junctions.

[1] T. Ng *et al.*, "Class 3 Semaphorin Mediates Dendrite Growth in Adult Newborn Neurons through Cdk5/FAK Pathway," *PLoS One*, vol. 8, no. 6, pp. 1–15, 2013, doi: 10.1371/journal.pone.0065572.

[2] X. Y. Yang, R. E. Stanley, A. P. Ross, A. M. Robitaille, J. A. Gray, and B. N. R. Cheyette, "Sestd1 Encodes a Developmentally Dynamic Synapse Protein That Complexes With BCR Rac1-GAP to Regulate Forebrain Dendrite, Spine and Synapse Formation," *Cereb. Cortex*, vol. 29, no. 2, pp. 505–516, Feb. 2019, doi: 10.1093/cercor/bhx333.

[3] E. Kaplan *et al.*, "ASCT1 (Slc1a4) transporter is a physiologic regulator of brain D -serine and neurodevelopment," *Proc. Natl. Acad. Sci.*, vol. 115, no. 38, pp. 9628–9633, Sep. 2018, doi: 10.1073/pnas.1722677115.

[4] I. Sahu, P. Nanaware, M. Mane, S. W. Mulla, S. Roy, and P. Venkatraman, "Role of a 19S Proteasome Subunit-PSMD10 Gankyrin in Neurogenesis of Human Neural Progenitor Cells," *Int. J. Stem Cells*, vol. 12, no. 3, pp. 463–473, Nov. 2019, doi: 10.15283/ijsc19007.

[5] M.-J. Xie *et al.*, "Phldb2 is essential for regulating hippocampal dendritic spine morphology through drebrin in an adult-type isoform-specific manner," *Neurosci. Res.*, vol. 185, no. 450, pp. 1–10, Dec. 2022, doi: 10.1016/j.neures.2022.09.010.

[6] A. Izmiryan, E. Peltekian, D. Paulin, Z. Li, and Z. Xue, "Synemin Isoforms in Astroglial and Neuronal Cells from Human Central Nervous System," *Neurochem. Res.*, vol. 35, no. 6, pp. 881–887, Jun. 2010, doi: 10.1007/s11064-009-0111-9.

[7] S. M. Sullivan *et al.*, "Cytoskeletal anchoring of GLAST determines susceptibility to brain damage: An identified role for GFAP," *J. Biol. Chem.*, vol. 282, no. 40, pp. 29414–29423, 2007, doi: 10.1074/jbc.M704152200.

[8] S. Schacke *et al.*, "Ezrin deficiency triggers glial fibrillary acidic protein upregulation and a distinct reactive astrocyte phenotype," *Glia*, vol. 70, no. 12, pp. 2309–2329, 2022, doi: 10.1002/glia.24253.

[9] M. Potokar, M. Morita, G. Wiche, and J. Jorgačevski, "The Diversity of Intermediate Filaments in Astrocytes," *Cells*, vol. 9, no. 7, p. 1604, Jul. 2020, doi: 10.3390/cells9071604.

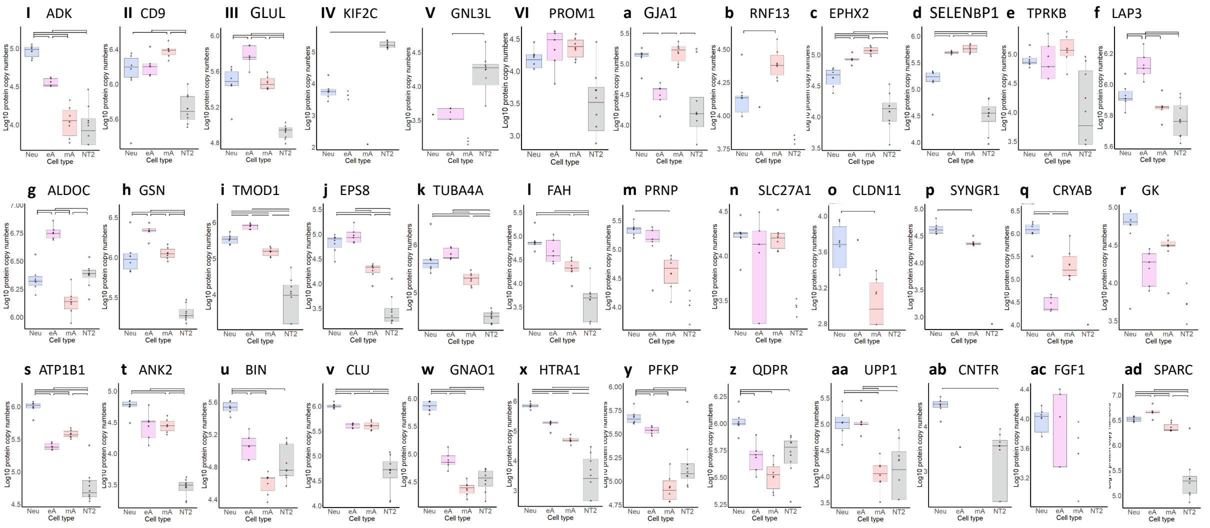

Fig. S9 **Levels of markers of mature astrocytes in Velloso's (I-VI)** (Velloso et al., 2022) **and Lattke's articles (a-ad)** (Lattke et al., 2021) **in examined cell types.** According to **Velloso** proteins I-III were upregulated and proteins IV-VI downreguletd in mature astrocytes. According to Lattke proteins a-ac were upregulated but protein ad dowregulated in mature astrocytes. Black lines indicate statistically significant differences as determined by ANOVA with post-hoc Tukey test, or, in cases of non-parametric conditions (checked by Shapiro-Wilk test), by Kruskal-Wallis and Mann-Whitney/Wilcoxon tests with Benjamini-Hochberg correction. In the event of missing values: if the proportion of missing values within a group is no more than 40%, then those missing values are replaced with the minimum value for that group. Box is signed when protein was identified in at least 60% samples in group

Lattke, M., Goldstone, R., Ellis, J. K., Boeing, S., Jurado-Arjona, J., Marichal, N., MacRae, J. I., Berninger, B., & Guillemot, F. (2021). Extensive transcriptional and chromatin changes underlie astrocyte maturation in vivo and in culture. *Nature Communications*, *12*(1), 4335. https://doi.org/10.1038/s41467-021-24624-5

Velloso, F. J., Shankar, S., Parpura, V., Rakic, P., & Levison, S. W. (2022). Neural Stem Cells in Adult Mammals are not Astrocytes. *ASN Neuro*, *14*, 175909142211347. https://doi.org/10.1177/17590914221134739

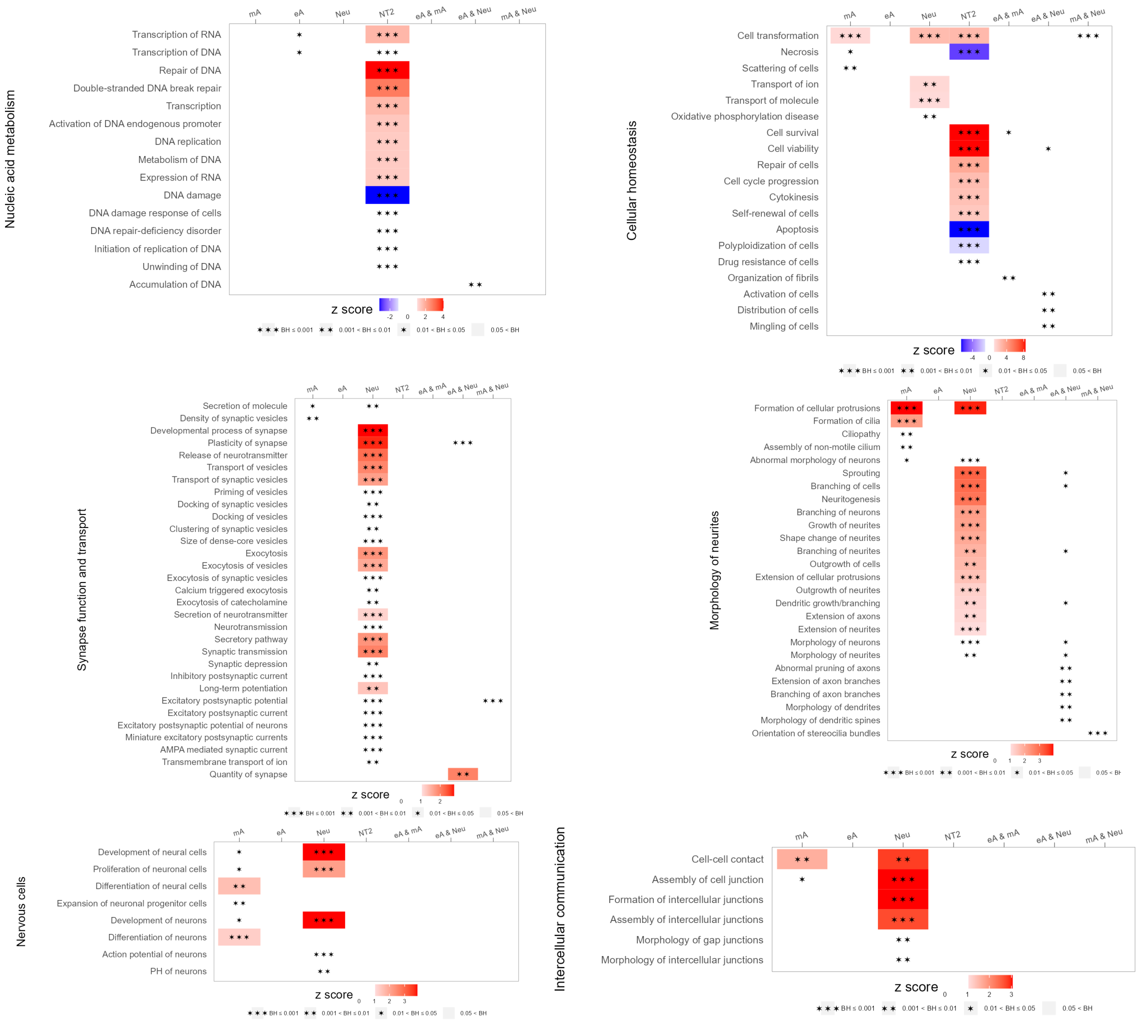

Fig. S10 **The cellular functions** obtained in the IPA (p-value with Benjamini-Hochberg correction <0.05) for proteins found to be unique to a given cell type or a pair of cell types

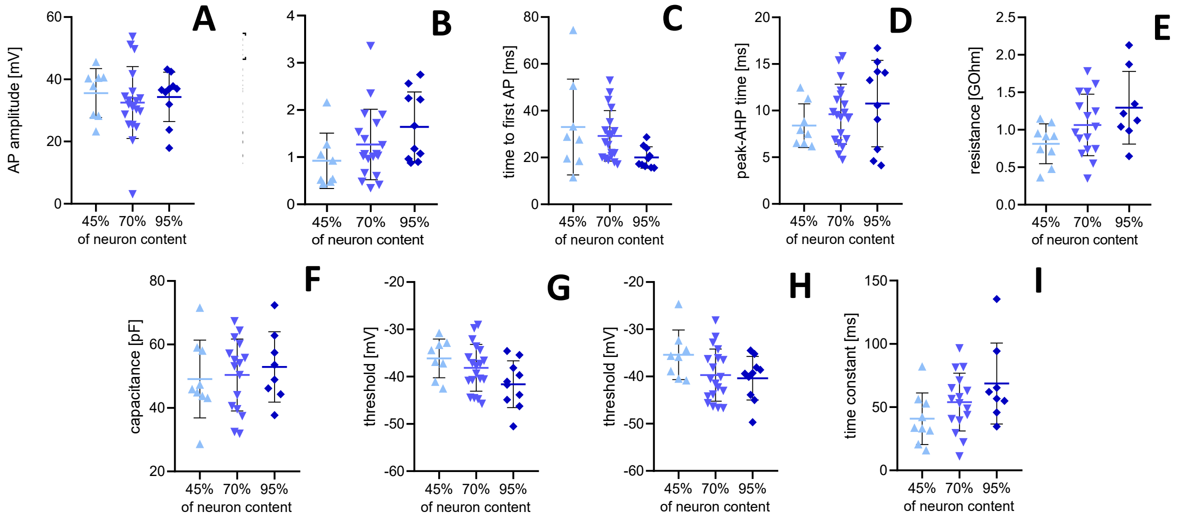

Fig. S11 **Electrophysiological properties of neurons:** amplitude of the first action potential induced by +80 nA pulse (**A**), time from 10-90% of the rising phase of the first action potential induced by +80 nA pulse (**B**), time to the first action potential induced by +80 nA pulse (**C**), time from peak to afterhyperpolarization of the first action potential induced by +80 nA pulse (**D**), neuronal membrane resistance (**E**), membrane capacitance (**F**), threshold for generating first action potential at a +80 nA current pulse (**G**), threshold for generating action potential measured from a current ramp (**H**), neuronal membrane time constant (**I**)

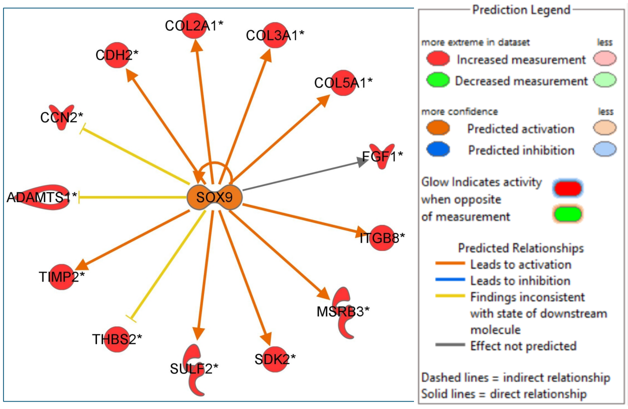

Fig. S12 IPA network for SOX9 as upstream regulator (p-value = 0.003, BH = 0.01, z-score = 1.6, bias) obtained in analysis of proteins identified with high confidence in eA cells but absent in NT2 cells. Noticed changes in level of effector proteins suggest upregulation of transcription factor, SOX9, in eA cells in relation to NT2 cells

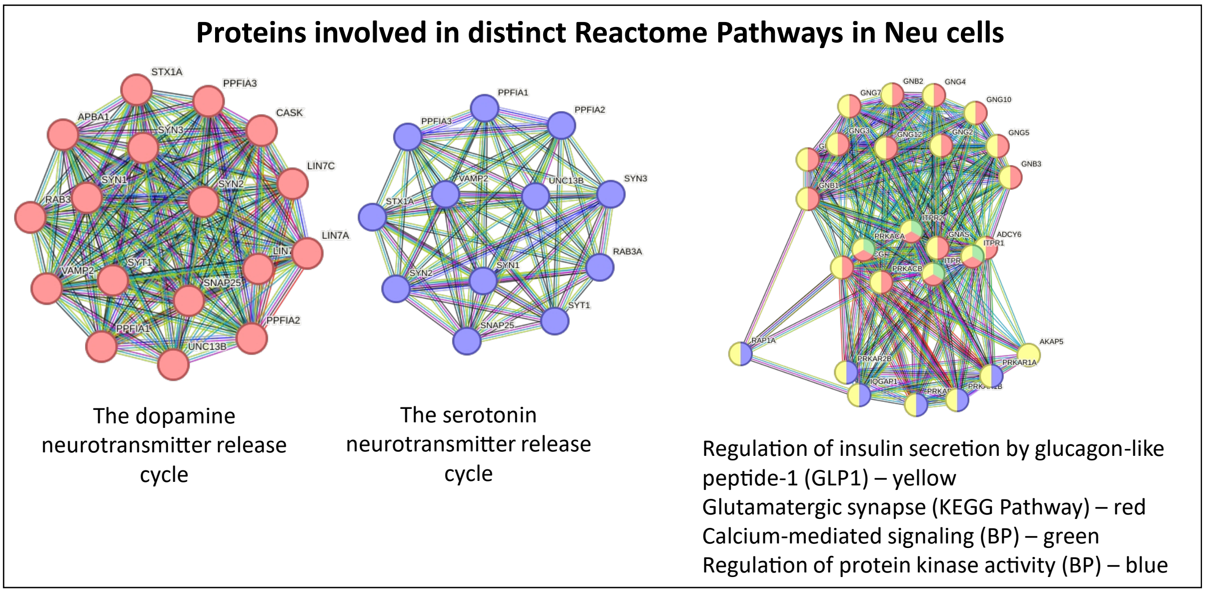

Fig. S13 Proteins involved in distinct Reactome Pathways in Neu cells obtainedwith FunRich and visualised with STRING: functional protein association networks (https://string-db.org).

GO enrichment analysis of proteins uniquely expressed in studied groups (i.e. present in at least 50% of the samples in each sample group, with the sum of sequenced peptides for a given protein being at least 130% of the number of samples in the group) using FunRICH (a p-value threshold with a Benjamini-Hochberg correction of less than 0.05 was applied; Table S5).

Among the statistically significant Reactome Pathways (RPs) unique to Neu cells and identified from all confidentially identified proteins in Neu, were the dopamine neurotransmitter release cycle and the serotonin neurotransmitter release cycle (Fig. S13). Indeed it has been demonstrated that under certain culturing conditions (with specific differentiation cocktails), neurons derived from NT2 cells can synthesize dopamine [1] or serotonin [2]. However, in our Neu culture, the amounts of tyrosine and tryptophan hydroxylases were below the detection limit of the mass spectrometer used. Nonetheless, the presence of many other proteins related to the dopamine and serotonin release cycles in neurons of the Neu culture confirmed their capability to synthesize these neurotransmitters.

Interestingly, another distinctive RP identified specifically in Neu cells was glucagon-like peptide-1 (GLP1) that modulates insulin secretion (R-HSA-381676). More detailed analysis of cellular functions of

the proteins within this pathway indicate the involvement of calcium-mediated signaling, glutamatergic synapse signaling and kinase activity (Fig. S13). GLP1, a peptide hormone and growth factor, has remarkable neuroprotective properties. GLP1 can be synthesized by a specific group of neurons, particularly within the brainstem. Receptors for GLP1 (GLP1R) are distributed throughout the central nervous system (CNS). Activation of GLP1R leads to various beneficial effects, including protection, regulation of calcium levels and ER stress, improvement in neuronal insulin sensitivity and energy metabolism, as well as an increase in the levels of BDNF and GDNF (glial cell line-derived neurotrophic factor) [3]. Analysis of canonical pathways using the IPA database for proteins uniquely expressed in Neu cells showed strong up-regulation of insulin secretion signaling pathway (Fig. 3B). Substantial quantities of insulin can be produced by both astrocytes and neurons, especially the GABAergic neurons. In neurons, insulin was shown to be released in an activity-dependent manner [4].

eA cells were particularly enriched in proteins involved in the following BPs: positive regulation of ATP-dependent activity, mitochondrial ATP synthesis coupled with electron transport, fatty acid beta-oxidation utilizing acyl-CoA oxidase, positive regulation of histone acetylation, positive regulation of RNA splicing, and the multivesicular body sorting pathway.
Both eA and mA cells showed a downregulation of SMAD2/3:SMAD4 transcriptional activity pathway. Meanwhile, mA cells contained proteins involved in the respiratory electron transport chain, fatty acid metabolic process, negative regulation of insulin receptor signaling pathway, positive regulation of protein catabolic process, positive regulation of microtubule polymerization but negative regulation of actin filament polymerization, intraciliary anterograde transport, and receptor-mediated endocytosis. Biological processes unique to NT2 cells were associated with DNA replication and transcription and the positive regulation of stem cell population maintenance.

The following molecular functions were characteristic for the studied cells: protein phosphatase activator activity and S100 protein binding (for eA); insulin receptor substrate binding (for mA); syntaxin-1 binding and phosphatidylinositol-3,5-bisphosphate binding (for Neu), and DNA binding and methylated histone binding (for NT2).

Proteins identified in eA cells were specifically localized to the cellular compartments such as the calcineurin complex and the multivesicular body membrane. Both eA and mA cells contained proteins involved in ciliary functions, with the ciliary tip present in both eA and mA cells. The BBSome was detected specifically in eA cells, while the intraciliary transport particle B was found in mA cells.

[1] L. Iacovitti, N. D. Stull, and H. Jin, “Differentiation of human dopamine neurons from an embryonic carcinomal stem cell line,” *Brain Res.*, vol. 912, no. 1, pp. 99–104, Aug. 2001, doi: 10.1016/S0006-8993(01)02723-8.

[2] C. Chu *et al.*, “TIAM2S as a novel regulator for serotonin level enhances brain plasticity and locomotion behavior,” *FASEB J.*, vol. 34, no. 2, pp. 3267–3288, Feb. 2020, doi: 10.1096/fj.201901323R.

[3] N. Reich and C. Hölscher, “The neuroprotective effects of glucagon-like peptide 1 in Alzheimer’s and Parkinson’s disease: An in-depth review,” *Front. Neurosci.*, vol. 16, no. September, pp. 1–55, Sep. 2022, doi: 10.3389/fnins.2022.970925.

[4] I. Pomytkin *et al.*, “Insulin receptor in the brain: Mechanisms of activation and the role in the CNS pathology and treatment,” *CNS Neurosci. Ther.*, vol. 24, no. 9, pp. 763–774, Sep. 2018, doi: 10.1111/cns.12866.
